# Discovery of an Adaptive Neuroimmune Response Driving Itch and Fast Tick Removal with Implications for Preventing Pathogen Transmission

**DOI:** 10.1002/advs.202517742

**Published:** 2026-01-27

**Authors:** Johannes S. P. Doehl, Tiago D. Serafim, Serena Doh, Charles S. Grugan, Eva Iniguez, Luana Rogerio, Ronja Frigard, Ranadhir Dey, Pedro Cecilio, Xinglong Gu, Pang‐Yen Tseng, Aline da Silva Moreira, Mahnaz Minai, James Oristian, Hans Ackerman, Steven Brooks, Caroline Percopo, Siu‐Ping Ng, Derron A. Alves, Lucas Tirloni, Jennifer M. Anderson, Adriana Marques, Fabiano Oliveira, Shaden Kamhawi, Daniel E. Sonenshine, José M. C. Ribeiro, Mark Hoon, Jesus G. Valenzuela

**Affiliations:** ^1^ Vector Molecular Biology Section Laboratory of Malaria and Vector Research National Institute of Allergy and Infectious Diseases National Institutes of Health Rockville Maryland USA; ^2^ Laboratory of Emerging Pathogens (LEP) Division of Emerging and Transfusion Transmitted Diseases (DETTD) Office of Blood Research and Review (OBRR) Center for Biologics Evaluation and Research (CBER) FDA Silver Spring Maryland USA; ^3^ Molecular Genetics Section National Institute of Dental and Craniofacial Research National Institutes of Health Bethesda Maryland USA; ^4^ University of Maryland School of Nursing Department of Pain and Translational Symptom Science Baltimore Maryland USA; ^5^ Infectious Disease and Pathogenesis Section Comparative Medicine Branch National Institute of Allergy and Infectious Diseases National Institutes of Health Rockville Maryland USA; ^6^ Physiology Unit Laboratory of Malaria and Vector Research National Institute of Allergy and Infectious Diseases National Institutes of Health Rockville Maryland USA; ^7^ Laboratory of Malaria and Vector Research National Institute of Allergy and Infectious Diseases National Institutes of Health Rockville Maryland USA; ^8^ Lyme Disease Studies Unit Laboratory of Clinical Immunology & Microbiology National Institute of Allergy and Infectious Diseases National Institutes of Health Bethesda Maryland USA; ^9^ Tick‐Pathogen Transmission Unit Laboratory of Bacteriology National Institute of Allergy and Infectious Diseases National Institutes of Health Rocky Mountain Laboratories Hamilton Montana USA; ^10^ Vaccine Research Center National Institute of Allergy and Infectious Diseases National Institutes of Health Bethesda Maryland USA; ^11^ Vector Biology Section Laboratory of Malaria and Vector Research National Institute of Allergy and Infectious Diseases National Institutes of Health Rockville Maryland USA

**Keywords:** adaptive immunity, ectoparasite, itch, neuroimmune response, tick removal, ticks

## Abstract

Acquired tick resistance (ATR) is well characterized in tick‐exposed animals, compromising tick fitness through antibody‐mediated activation of basophils. Yet, anti‐tick vaccines inducing ATR have had limited success. Here, we describe a neuroimmune event preceding ATR that leads to rapid host‐mediated tick removal. Tick‐sensitized guinea pigs mechanically remove ticks within 3–6 h via an acquired neuroimmune‐induced itch response that correlates with increased dermal expression of itch‐associated genes and skin infiltration by CD3^+^T cells and Iba‐1^+^ macrophages, independent of IgG and IgE antibodies. Inhibiting the development of acquired T cell memory by averting naive lymphocyte egress from lymph nodes with the sphingosine‐1‐phosphate receptor modulator FTY720, before tick sensitization, prevents Iba‐1^+^ macrophage and CD3^+^ T cell infiltration to the tick bite site and abrogates scratching and tick removal. This neuroimmune response is independent of Trpv1 as tick‐sensitized guinea pigs treated with the Trpv1 agonist, resiniferatoxin, remove ticks effectively. Strikingly, prior exposure to a single tick is sufficient to generate a fast and active itch‐induced tick removal (IITR) that is observed even in tick‐attached sites distant from the location of previous tick exposure. IITR represents a novel approach to tick‐borne disease prevention through early tick detection and fast removal.

## Graphical abstract

Itch‐induced tick removal (IITR): An acquired neuroimmune mechanism, itch‐induced tick removal, develops after repeated tick exposure, mobilizing T cells and macrophages at the tick bite site to trigger a rapid scratching response that facilitates timely tick removal within a critical window that precedes the transmission of many tick‐borne pathogens.

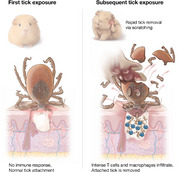

## 1 Introduction

Ticks are the most important arthropod vectors of disease in the United States, and they transmit a variety of pathogens, including bacteria, viruses, and protozoa [1, 2, 3, 4]. The tick *Ixodes scapularis* (black‐legged tick) is considered the most relevant tick species in the United States, transmitting human pathogens that cause Lyme disease, human granulocytic anaplasmosis, and babesiosis among other infectious diseases [3, 4].

Most tick pathogens are transmitted slowly, requiring between 24 to 48 h of tick attachment to the host [5]. This offers a window of opportunity to target the tick before pathogens are transmitted. To date, anti‐tick vaccines have focused on identifying tick‐derived molecules, mostly salivary or gut proteins, that can prevent tick feeding or kill the tick before pathogens are transmitted [6].

The roots of anti‐tick vaccines come from the observation that animals exposed multiple times to tick bites become resistant to further tick infestations [7]. This phenomenon, named acquired tick resistance (ATR), results in reduced tick engorgement, reduced tick egg‐masses, premature tick detachment, and death of the biting tick [7, 8, 9]. The hallmark of ATR is the presence of basophils at the tick bite site and the requirement for antibodies, and both have been associated with poor tick feeding or compromised tick fitness observed around days 3–4 post attachment [10, 11]. Attempts to accelerate this response by developing an ATR‐mediated anti‐tick vaccine that prevents tick feeding and achieves tick detachment by or before 48 h have only been partially successful, and current vaccines that compromise tick feeding and accelerate tick detachment do so only at 72 h post tick bite [6, 12, 13]. Unfortunately, this is too late to prevent transmission of most tick‐borne pathogens.

Notably, tick loss at 24–48 h has been observed in the gold standard model of multiple tick exposures in guinea pigs (GPs) whose mobility was unimpeded [14]. This strongly suggests that there may be another mechanism, independent of poor tick feeding or loss of tick fitness, that is responsible for the early loss of ticks at 24–48 h in tick‐sensitized animals. A study identified an intriguing correlation between individuals who reported repeated episodes of itch at tick‐bite sites and a three‐fold reduction in Lyme disease incidence, compared to those who do not feel the itch [15]. This led us to hypothesize that early tick loss in tick‐sensitized animals may be mediated by itch through a rapid host‐mediated mechanism that precedes ATR. Many biting ectoparasites are known to induce itch in afflicted hosts by cutaneous hypersensitivity. Insect bites are thought to mediate itch through a type I hypersensitivity response, while arachnids, like mites (e.g., scabies), can induce itch through Type I and type IV hypersensitivity responses [10, 16, 17]. Much is known about itch detection, including that itch and pain are separate sensations, and that pruritogens are detected by transcriptionally distinct sensory nerves that transmit signals through specialized neural circuits [18, 19, 20]. In mice, the main model for itch research, three sensory neuron types, NP1, NP2, and NP3, detect itch, with NP3 neurons responsible for inflammatory itch [21, 22]. NP2 and NP3 neurons express Trpv1 and several itch receptors, including histamine receptors. NP2 neurons express Mrgpra3, while NP3 neurons express cytokine receptors such as OSM, IL‐31, and the cysteinyl leukotriene receptor, which heighten their activity [22, 23]. NP3 neurons also utilize the neurotransmitter Nppb, which activates Npr1‐spinal cord neurons [24]. Many of the key signaling components of NP3 are also present in classes of human DRG‐neurons, but differences in expression patterns of some NP3 key genes occur between species [25, 26, 27]. In guinea pigs, however, the neurons responsible for itch remain unknown [28].

Here, we show experimental evidence that bites from *Ixodes scapularis* (black‐legged tick) nymphs induce an adaptive protective itch/scratch response in *Cavia porcellus* GPs, which results in rapid tick removal to subsequent tick exposures. The host immune response is acquired in only 5–6 days after the initial bite and is dependent on an effective adaptive lymphocyte‐mediated cellular immune response that triggers a Trpv1‐independent itch pathway and a targeted scratch response within hours after biting. The itch/scratch response is localized to the tick bite site and results in the mechanical removal of ticks. The rapid occurrence of this response, enabling GPs to remove ticks as early as 3 h after bite, provides opportunities to prevent the transmission of important tick‐borne pathogens, including *Borrelia, Anaplasma, Ehrlichia, Babesia*, and *Rickettsia* species.

## 2 Results

### 2.1 Scratching Mediates Early Tick Removal Without Affecting Tick Fitness

**FIGURE 1:**
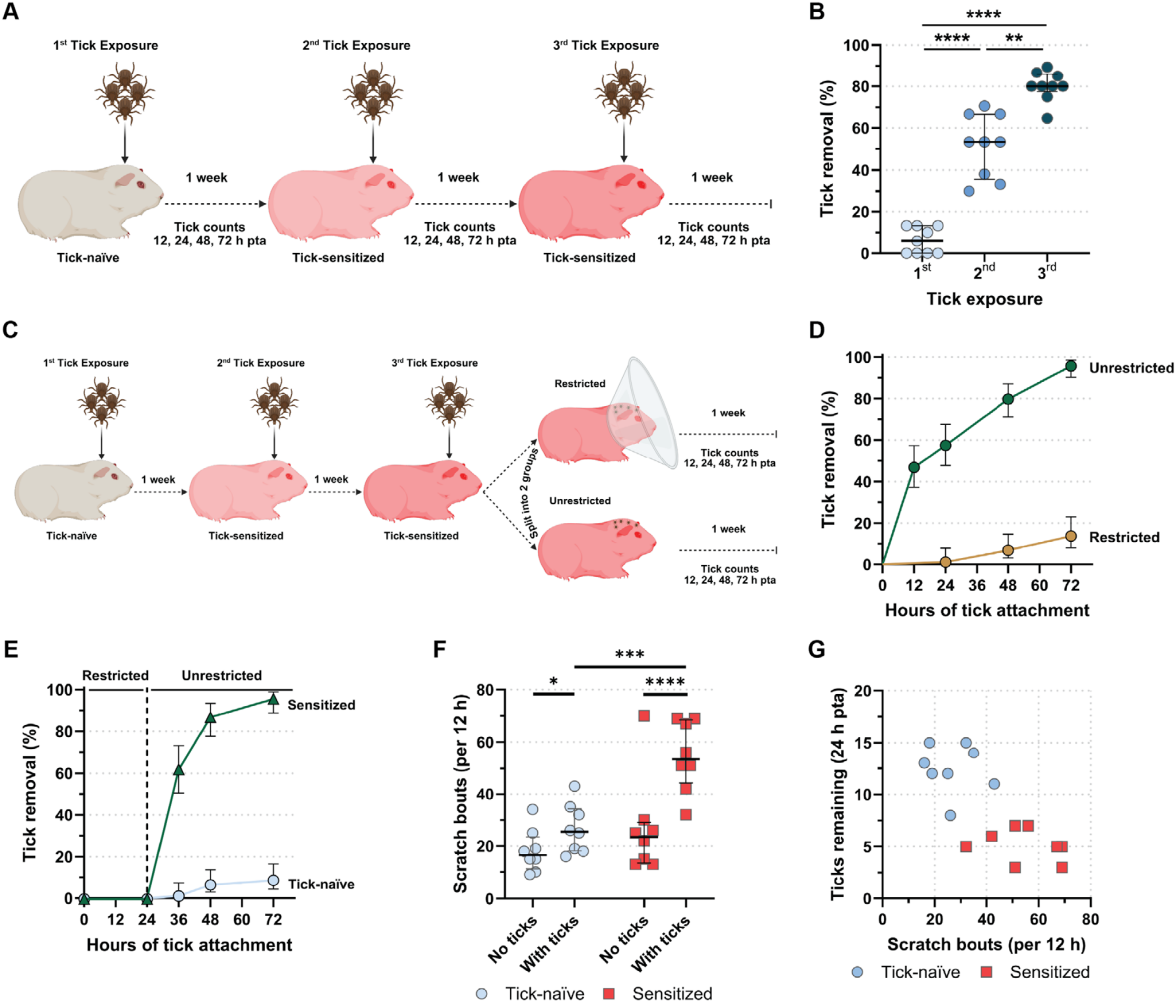
Local scratching mediates rapid tick removal. (A) Repeated tick exposure experimental design (Created in BioRender: https://BioRender.com/47zaovj). (B) Tick removal in percent of total ticks placed per guinea pig at 48 h post tick attachment (pta) after repeated weekly tick exposures (*N* = 15 ticks/guinea pig (GP); *N* = 9 GPs per exposure; same nine GPs reused in each exposure). Pairwise estimated marginal means: first vs second: *p* < 0.0001; first vs third: *p* < 0.0001; second vs third: *p* = 0.004. Median ± IQR shown. (C) Experimental concept of D (Created in BioRender: https://BioRender.com/2jxmm9k). (D,E) Smoothed, inverted Kaplan–Meyer plot showing probability of tick removal (%) in (D) restricted (total *N* = 86 ticks) and unrestricted (total *N* = 94 ticks) tick‐access in 5x‐exposed GPs (*N* = 7/group), and (E) tick‐naive (*N* = 93 ticks) and 6x‐exposed (*N* = 68 ticks) GPs (*N* = 6/group) after E‐collar‐removal 24 h pta. (D,E) Analysis by Peto & Peto modification of the Gehan–Wilcoxon test: (D) *p* < 0.0001, (E) *p* < 0.0001. 95% CI is shown. (F) Scratch bouts/12 h in tick‐naive and tick‐sensitized GPs (*N* = 8/group). The same GPs were analyzed first without, then with ticks attached (total *N* = 181 ticks and *N* = 169 ticks, respectively). Pairwise estimated marginal means: tick‐naive without and with ticks: *p* = 0.0231; tick‐sensitized without and with ticks: <0.0001, tick‐naive vs tick‐sensitized guinea pigs both with ticks: *p* < 0.0001. Median ± IQR shown. (G) Scatter plot of scratch bouts/12 h against ticks remaining (24 h pta) in tick‐naive and tick‐sensitized GPs (*N* = 8/group). One‐way MANOVA: *p* < 0.0001. ns > 0.05, ^*^ ≤0.05, ^**^ <0.01, ^***^ <0.001, ^****^ <0.0001. For detailed statistics see supplementary statistics report.

We subjected guinea pigs (GPs, *Cavia porcellus*) to a series of weekly‐spaced, capsule‐free nymphal *Ixodes scapularis* tick exposures to observe the frequency of tick loss over the course of 72 h post tick attachment (Figure 1A). We found that during the second and third tick exposures, GPs lost a median of 53% and 80% of ticks within 48 h post tick attachment (pta), respectively, compared to only 6% loss for tick‐naive animals during the first tick exposure (Figure 1B) similarly to what has been previously reported [7, 29, 30]. To test if this tick loss is due to a direct animal tick removal as suggested by Burke et al. (2005) and not to ATR‐driven immunity directly preventing tick feeding, we used Elizabethan collars on tick‐sensitized GPs to prevent animals from reaching the attached ticks (Figure 1C) [15]. Surprisingly, tick loss by sensitized GPs was abrogated by using Elizabethan collars (Figure 1D), suggesting that ticks are actively removed by the GPs. Further supporting this hypothesis, tick removal was reconstituted in tick‐sensitized animals only after Elizabethan collars were removed (Figure 1E).

**FIGURE 2:**
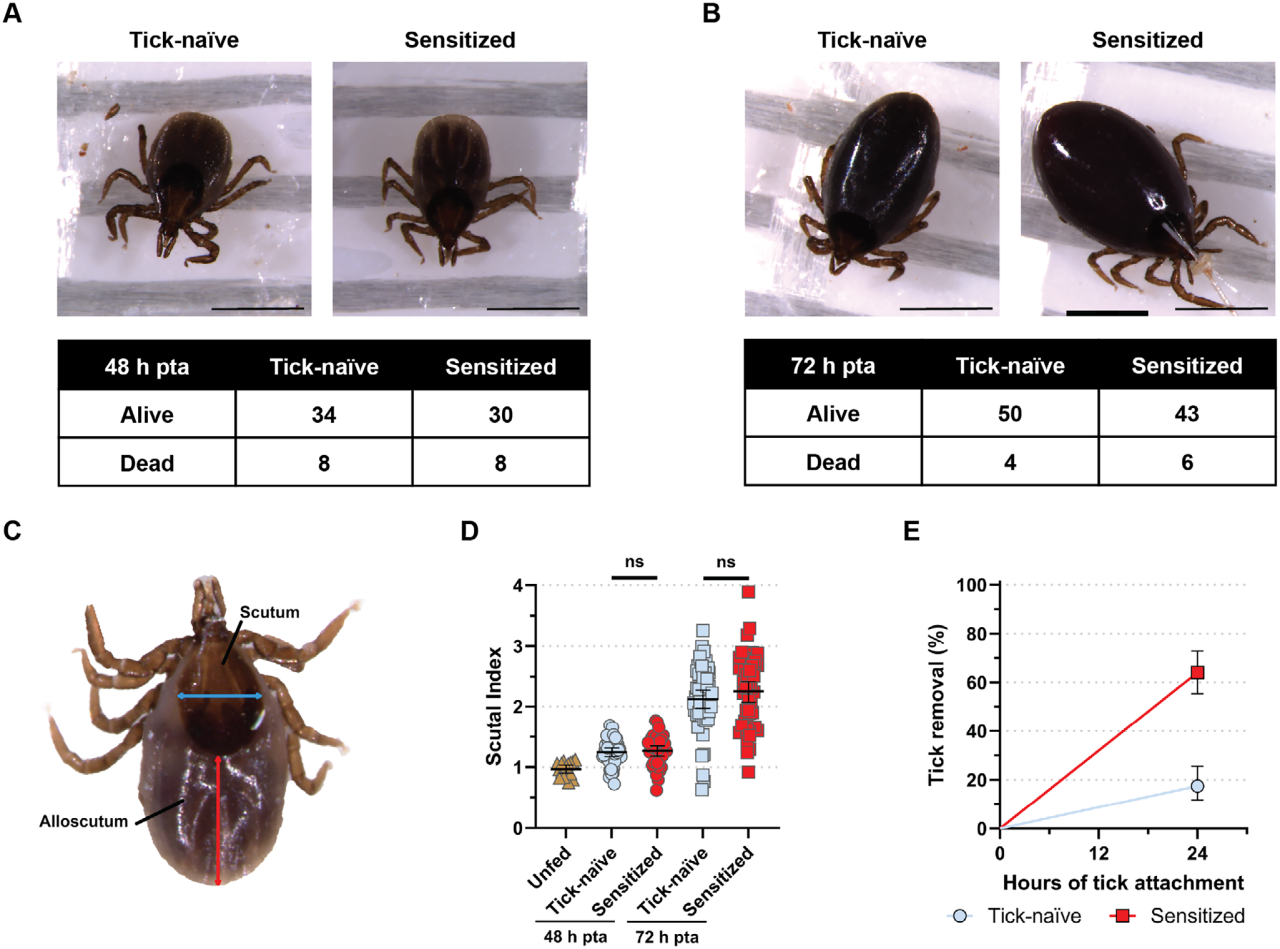
No tick feeding impairment by 48 and 72 h pta on sensitized guinea pigs. (A,B) Images of ticks fed on sensitized and tick‐naive guinea pigs (GPs) with a respective cross table of live/dead tick counts at 48 and 72 h post tick attachment (pta). Cross table analysis by (A) Chi^2^ test (*p* = 1, *N* = 42 [tick‐naive], *N* = 38 [Sensitized]), and (B) Fisher's Exact test (*p* = 0.5124, *N* = 54 [tick‐naive], *N* = 49 [Sensitized]). (C) The scutal index is a ratio produced by dividing the alloscutum's length by the scutum's width. (D) Scatter plot: Scutal index values of nymphal ticks fed on tick‐naive (48 h pta: *N* = 42, 72 h pta: *N* = 54) and sensitized (48 h pta: *N* = 38, 72 h pta: *N* = 49) GPs, or unfed (*N* = 16). Two‐way robust ANOVA (excluding unfed): Sensitization state (tick‐naive versus tick sensitized) *p* = 0.3229, Time (48 h pta versus 72 h pta) *p* < 0.0001. Mean ± 95% CI shown. (E) Smoothed, inverted Kaplan–Meyer plot showing probability of tick removal (%) during a first (Tick‐naive, *N* = 120 ticks) and a fourth (Sensitized, *N* = 114 ticks) tick‐exposure of GPs (*N* = 8/group) at 24 h pta. Analysis by Peto & Peto modification of the Gehan‐Wilcoxon test: *p* < 0.0001. 95% CI shown. ns > 0.05, ^*^ ≤0.05, ^**^ <0.01, ^***^ <0.001, ^****^ <0.0001. For detailed statistics see supplementary report.

To directly test if tick removal is the result of scratching by tick‐sensitized GPs, we used an automated behavior observational research system (EthoVision, Noldus) to measure scratch activity. Compared to tick‐naive animals, a significantly greater increase in scratch bouts was observed at the site of tick attachment after the third tick exposure, in contrast to basal levels of scratching activity in animals with no ticks in both tick‐sensitized and tick‐naive GPs (Figure 1F). Furthermore, an increase in the total scratch bouts correlated with a decrease in the number of attached ticks by 24 h pta (Figure 1G). As scratching is a proxy for itching, our results suggest that itch is an acquired response to ticks, and it is responsible for the early tick removal in tick‐sensitized GPs [31, 32]. Because the observed itch/scratch response is localized to the tick bite site and results in the mechanical removal of ticks, we named this response Itch‐Induced Tick Removal or IITR. Of note, at 48 and 72 h pta, tick survival and tick feeding/engorgement success, measured by the tick scutal index, were comparable between the restricted 4‐times tick‐sensitized and tick‐naive groups (Figure 2A–D). This indicates that this early tick removal is not due to obstruction of tick feeding.

### 2.2 IITR is Associated with a Skin Cellular Infiltrate at the Bite Site That is Antibody Independent

**FIGURE 3:**
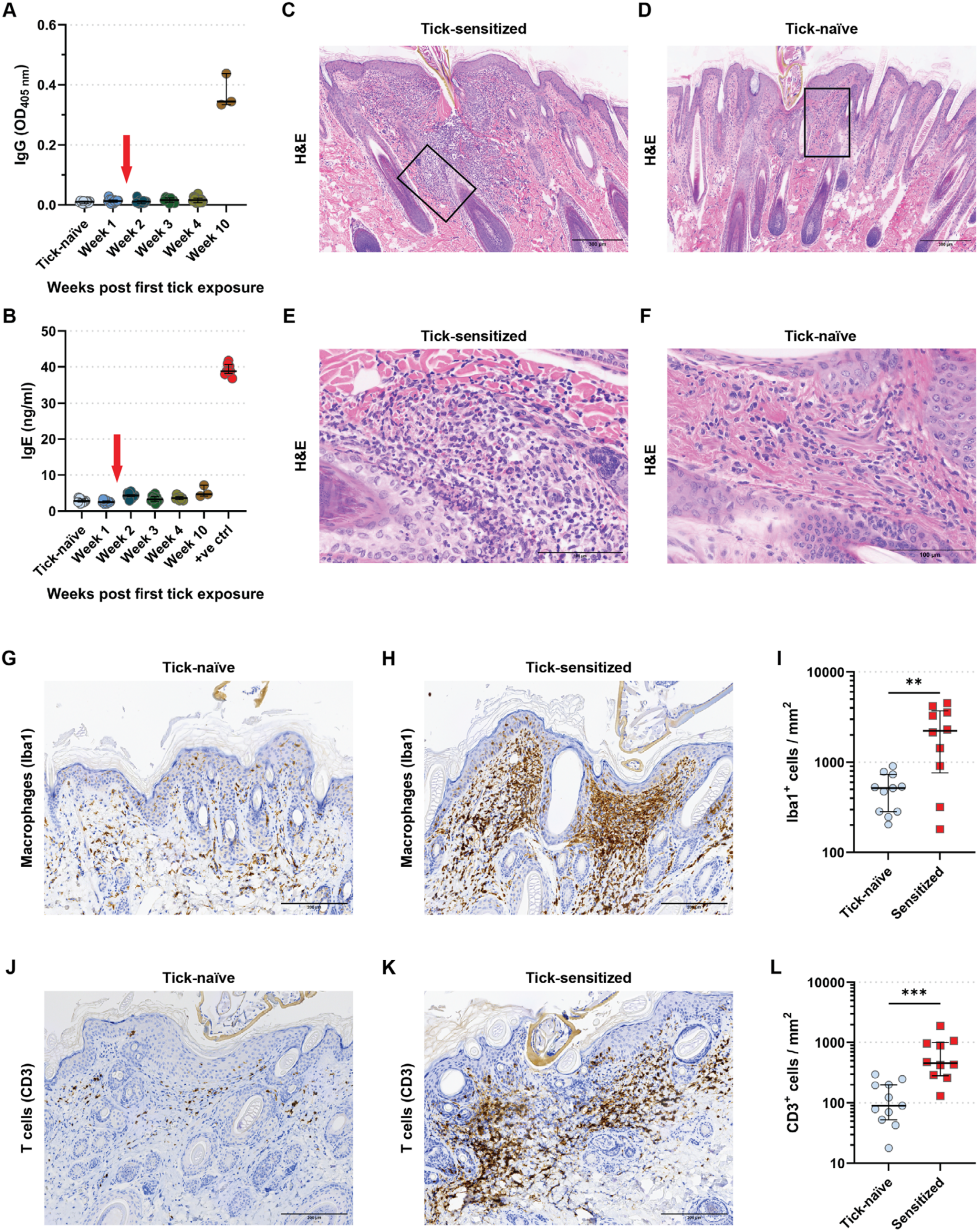
Rapid tick removal is associated to the recruitment of T cells and macrophages but not to IgG or IgE. (A,B) ELISA of anti‐tick saliva (A) IgG (Tick‐naive, Week 1, Week 2: *N* = 15, Week 3: *N* = 9, Week 4: *N* = 14, Week 10: *N* = 3) and (B) total IgE (Tick‐naive, Week 1, Week 2: *N* = 15, Week 3, Week 4: *N* = 9, Week 10: *N* = 3, +vs ctrl: *N* = 8) titers in guinea pig (GP) sera pre‐ and up to 10 weeks post first tick‐exposure. Pairwise estimated marginal means: (A) All other time‐points versus Weeks 10: *p* < 0.0001; (B) All time‐points versus positive control (+ve ctrl): *p* < 0.0001. Median ± IQR shown. (C,D) Representative H&E skin cross‐sections (>|5µm|<) of tick bite sites 24 h post tick attachment (pta) in (C) first‐time exposed tick‐naive and (D) three times weekly tick‐sensitized Guinea pigs. (C) Mild epidermal hyperplasia with a minimal dermal infiltrate. (D) Significant focally extensive dermal inflammation consisting primarily of a mononuclear cell infiltrate surrounding and separating adnexal structures. Scale bar: 300 µm. 10x (E,F) Enlarged section from (C,D) to illustrated (E) absence and (F) presence of eosinophils in tick bite sites. Scale bar: 100 µm. (G,H,J,K) IHC of skin biopsies from tick‐bite sites collected at 48 h post tick attachment (pta) from tick‐naive (G,J) and tick‐sensitized (H,K) GPs probed with anti‐Iba‐1 (macrophages; G,H) or anti‐CD3 (T cells; J,K) antibodies. Scale bar: 200 µm. (I,L) Scatter plot: Counts of (I) Iba‐1^+^ (macrophages), and (L) CD3^+^ (T cells) cells in skin sections of biopsies collected at 48 h pta from the first exposure of tick‐naive (*N*=11 sections), and the third exposures of tick‐sensitized (*N*=10 sections) GPs. Analyzed by Mann‐Whitney U test: (I) *p* = 0.0079 and (L) p=0.0002. Median ± IQR shown. ns > 0.05, ^*^ ≤0.05, ^**^ <0.01, ^***^ <0.001, ^****^ < 0.0001. For detailed statistics see supplementary report.

Given our findings that early tick removal is induced by itch and is an acquired response developed after one tick exposure (priming event), we reasoned that itch may be mediated by an adaptive immune response. Scratching is classically associated with IgE and IgG responses and activation of basophils and mast cells [33, 34]. Surprisingly, anti‐tick saliva IgG and total IgE levels in serum remain low in GPs up to week 4 and 10 pta, respectively, despite a second, third and fourth tick sensitization events on a weekly basis, when IITR responses are fully developed (Figure 3A,B), indicating that IITR responses are independent of anti‐tick saliva IgG and total IgE antibody levels.

We next investigated the recruitment of immune cells to the tick‐bite site in the skin of tick‐naive and three‐times tick‐sensitized GPs at tick bite sites. At 24 h pta, tick‐sensitized GPs exhibited a strong cellular infiltration (Figure 3C) that was absent in tick‐naive animals (Figure 3D) at the tick‐bite site. A few eosinophils were observed in the superficial and mid dermis of tick‐sensitized GPs (Figure 3E) and rarely observed in tick‐naive GPs (Figure 3F). The presence of mast cells was at base levels in both tick‐sensitized and tick‐naive GPs (Figure 3C–F). The skin infiltrate from tick‐sensitized GPs consisted of inflammatory cells, including Iba‐1^+^ macrophages (Figure 3G–I) and CD3^+^ T cells (Figure 3J–L) that were significantly increased compared to tick‐naive GPs. This cellular infiltration in tick‐sensitized animals began as early as 6 h pta and steadily increased at 12, 24, and 48 h pta (Figure S1).

### 2.3 IITR is Dependent on the Adaptive Cellular Immune Response to Tick Bites

**FIGURE 4:**
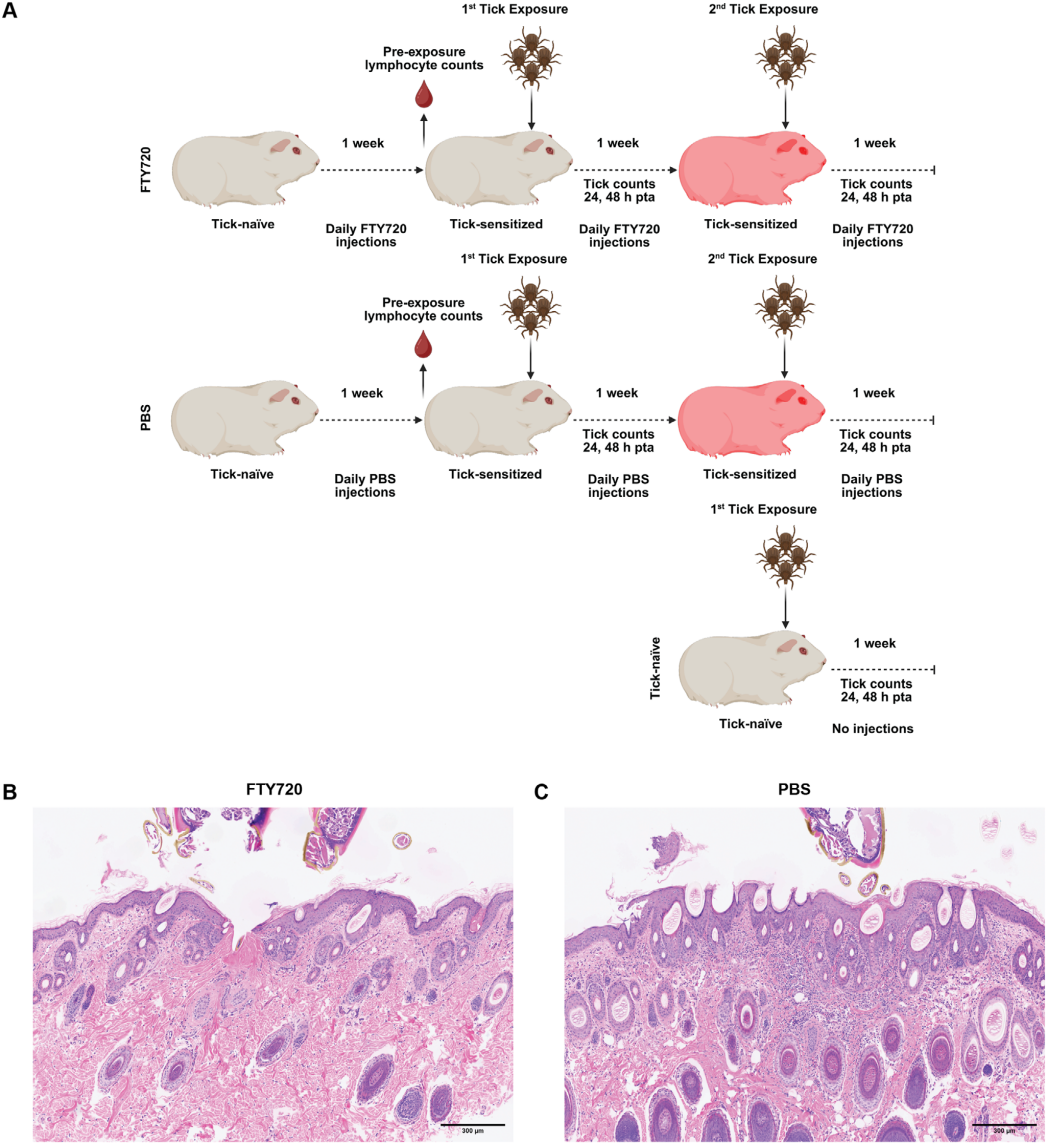
(A) FTY720 experimental design (Created in BioRender: https://BioRender.com/rduvqd2). Guinea pigs (GPs, *N* = 10/group) were either FTY720‐ or PBS‐treated and then exposed two times to 15 ticks each. First‐time exposed tick‐naive GPs were used as a tick attachment control. (B,C) Representative H&E skin cross‐sections (>|5µm|<) of a dermal biopsy at tick bite sites 48 h pta during the second tick exposure. 10x. (B) FTY720‐treated GP showing mild epidermal hyperplasia and dermal edema but a minimal dermal infiltrate reminiscent of a first‐time infested tick‐naive GP. (C) PBS‐treated GP showing significant dermal inflammation and epidermal hyperplasia.

**FIGURE 5:**
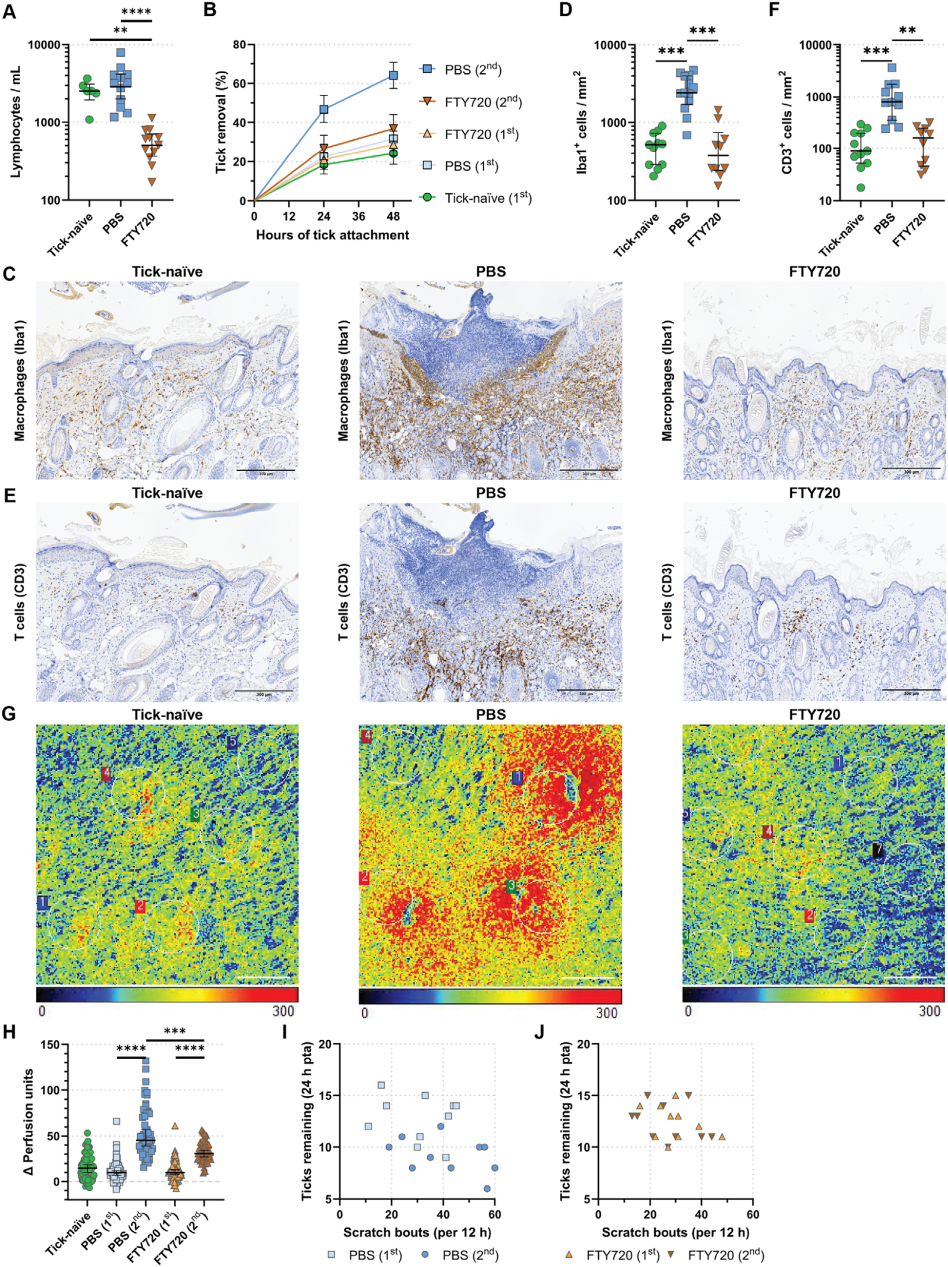
Rapid Tick Removal is dependent on a host adaptive immune response. (A) Scatter plots: Lymphocyte counts per milliliter of blood 5 days post FTY720 treatment initiation (tick‐naive: *N* = 6 guinea pigs (GPs), PBS, FTRY720: *N* = 12 GPs). Analyzed by Dunn's test: FTY720: Pre‐treatment versus Pre‐infestation: *p* = 0.0005; Pre‐treatment versus post‐infestation: *p* > 0.0001. Median ± IQR shown. (B) Smoothed, inverted Kaplan–Meyer plot showing probability of tick removal (%) during a first exposure of tick‐naive, and a first and second exposures of PBS‐ or FTY720‐treated GPs at 24 and 48 h post tick attachment (pta), *N* = 195 ticks/group; *N* = 13 GPs/group. Analysis by Cox Proportional Hazards Model: Tick‐naive versus PBS (second tick exposure): *p* = 0.0001, versus PBS (second tick exposure): *p* < 0.0001; FTY720 (first tick exposure) versus PBS (second tick exposure): *p* = 0.0012; FTY720 (second tick exposure) versus PBS (second tick exposure): p=0.0352. 95% CI is shown. (C,E) IHC of skin biopsies from tick‐bite sites collected at 48 h pta of tick‐naive and tick‐sensitized (PBS‐ or FTY720‐treated) GPs probed with (C) anti‐Iba‐1 (macrophages) or (E) anti‐CD3 (T cells) antibodies. Scale bar: 300 µm. (D,F) Scatter plots: Counts of (D) Iba‐1^+^ (macrophages), and (F) CD3^+^ (T cells) cells in skin sections of biopsies collected at 48 h pta from the first exposure of tick‐naive (*N* = 11 sections, either), and the second exposures of PBS‐ (*N* = 13 and *N* = 11sections, respectively) or FTY720‐treated GPs (*N* = 10 sections, either). Analyzed by Dunn's test: (D) second tick exposure of FTY720‐ versus PBS‐treated GPs: *p* = 0.0001, first tick exposure of tick‐naive versus second tick exposure of PBS‐treated GPs: *p* = 0.0003; and (F) second tick exposure of FTY720‐ versus PBS‐treated GPs: *p* = 0.0009, first tick exposure of tick‐naive versus second tick exposure of PBS‐treated GPs: *p* = 0.0003. Median ± IQR shown. (G,H) Laser speckle measurements of tick‐bite sites of tick‐naive and tick‐sensitized (PBS‐ or FTY720‐treated) GPs. (G) Perfusion images. Blue (0) = no signal, red (300) = high signal. Scale bar, 2 mm. (H) Scatter plot: Delta Perfusion units (tick‐bite site signal—image background signal). Tick‐naive, PBS (first tick exposure): *N* = 73; PBS (second tick exposure): *N* = 48; FTY720 (first tick exposure): *N* = 72; FTY720 (second tick exposure): *N* = 62. Pairwise estimated marginal means: PBS (first versus second tick exposure): *p* < 0.0001, FTY720 (first versus second tick exposure): *p* < 0.0001, second tick exposure (FTY720 versus PBS): *p* = 0.0006. Median ± IQR shown. (I,J) Correlation scatter plot of total scratch bouts/12 h versus total ticks remaining at 24 h pta of first and second time exposed GPs (*N* = 10/group). (I) Mock‐treated (PBS) and (J) FTY720‐treated. One‐way mixed MANOVA: (I) *p*<0.001; (J) *p* = 0.069. ns > 0.05, ^*^ ≤0.05, ^**^ <0.01, ^***^ <0.001, ^****^ <0.0001. For detailed statistics see supplementary report.

To directly evaluate whether IITR is dependent on the acquired cellular immune response observed in tick‐sensitized GPs, we used the immunosuppressor FTY720 (Fingolimod). FTY720, a sphingosine‐1‐phosphate receptor modulator, prevents the egress of naive lymphocytes from the lymph nodes, effectively depleting them from circulation and consequently preventing the development of acquired T cell memory immune responses [35]. GPs were treated daily with FTY720 starting 1 week prior to the first tick exposure and continuing throughout the first and the second tick exposures (Figure 4A). Consistent with its known effects, we found that FTY720‐treated sensitized GPs had a significantly reduced number of circulating lymphocytes compared to sensitized PBS‐treated or untreated tick‐naive GPs prior to the first tick exposure (Figure 5A). Notably, during a second tick exposure, FTY720‐treated GPs failed to show significant tick removal while the PBS‐treated GPs removed ticks over 48 h pta (*p* < 0.0001; Figure 5B). Loss of IITR correlated to an overall reduction of the dermal cellular infiltrate in FTY720‐treated (Figure 4B) compared to PBS‐treated (Figure 4C) GPs in twice sensitized animals at 48 h pta. Specifically, treatment with FTY720 significantly decreased the recruitment of Iba‐1^+^ macrophages (Figure 5C,D, FTY720), and CD3^+^ T cells (Figures 5, E and F, FTY720) to the tick bite site in comparison to PBS‐treated animals (Figure 5C–F, PBS). Furthermore, using laser speckle as a proxy for local skin cellular recruitment, we observed that continuous treatment of FTY720 abolished the robust increase in skin blood flow or cell recruitment to the tick bite site (Figure 5G,H, FTY720) observed in PBS‐treated GPs (Figure 5, G and H, PBS). The high level of cellular recruitment corresponded to an increase in scratch bouts and in tick removal during the second tick exposure among control PBS‐injected animals (Figure 5I PBS second). By contrast, FTY720‐treated GPs showed fewer scratching bouts upon a second tick exposure, corresponding to less effective tick removal (Figure 5J, FTY720 second). These data indicate that IITR relies on a lymphocyte‐mediated acquired immune response to ticks that resembles, in kinetics and cell recruitment, a Type IV delayed hypersensitivity response [36]. To further test if FTY720 has no off‐target effect and works mostly on naive lymphocytes, GPs previously exposed to tick bites (tick‐sensitized GPs) were treated with FTY720 or with PBS (control). The hypothesis was that FTY720 should not have any effect on animals that were already pre‐exposed to ticks, because FTY720 does not act on immune memory cells. Therefore, any observed effect would likely be due to innate cells (i.e., an off‐target effect). We found no difference between pre‐exposed animals treated with FTY720 and those treated with PBS, both groups removed ticks effectively by 48 h (Figure S2), strongly suggesting that FTY720 has no off‐target effect and acts only on naive lymphocytes, not on memory or other adaptive lymphocytes.

### 2.4 IITR is Independent of the Trpv1 Pathway

**FIGURE 6:**
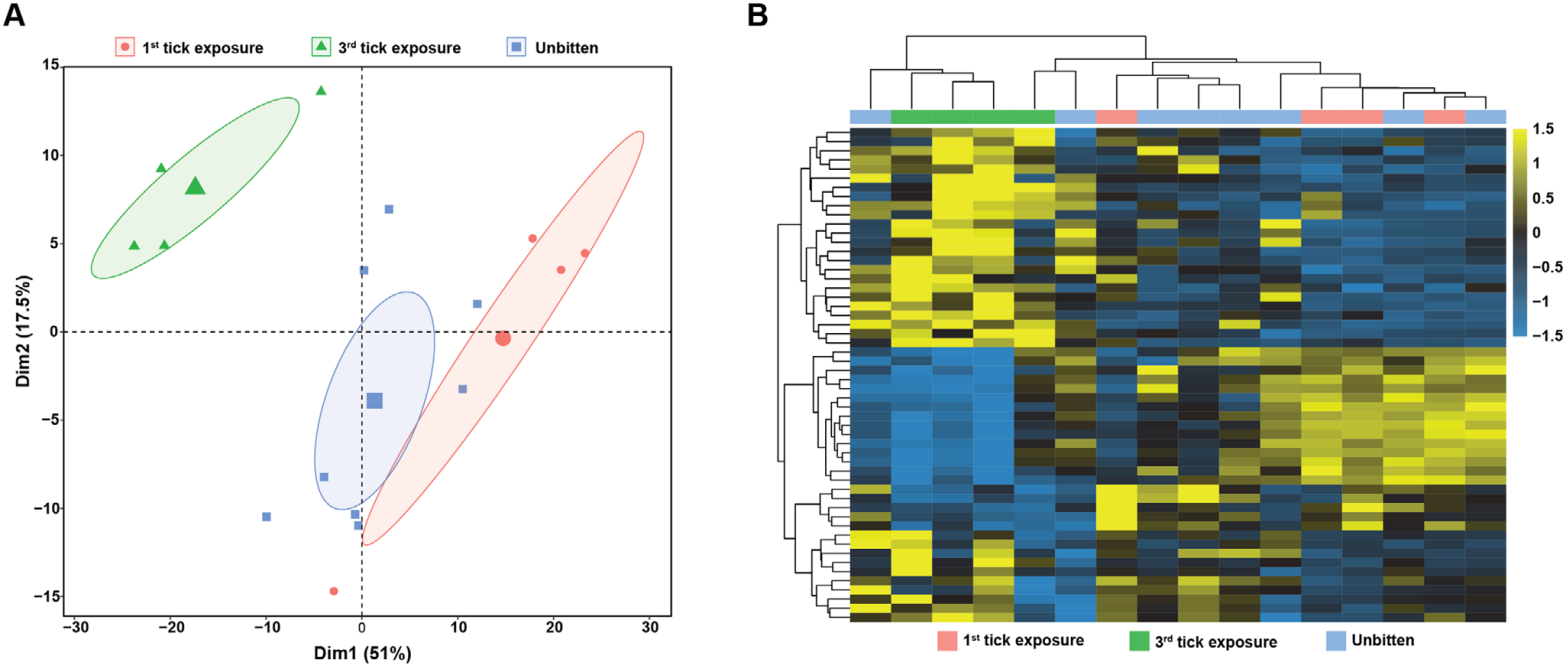
Bulk RNAseq show clear distinction in gene expression in tick‐sensitized bitten skin. RNAseq analysis of 3 mm skin biopsies from tick bite sites during a first (*N* = 4) and third (*N* = 4) tick exposure, and unbitten skin (*N* = 8). (A) PCA plot. (B) heatmap of the top 54 differentially expressed genes, either upregulated or downregulated, after applying a ± 3 log2 fold change cutoff.

Having established that IITR is evoked by itch, we wanted to identify potential itch signatures in tick‐sensitized GPs. To achieve this, we analyzed the differential expression of genes in skin cells from GPs sensitized once or three times to ticks (Figure 6). Principal component analysis distinguished three times tick‐sensitized GPs from both unbitten and once‐exposed GPs (Figure 6A). Further, we noted differential expression of genes and an increased expression of molecules known to modulate the itch response, including defensins, IL13, and OSM, in three times tick‐sensitized GPs compared to unbitten and once‐exposed GPs (Figure 6B and Table 1) [23].

**TABLE 1:** Upregulated skin transcripts at the tick‐bite site of tick‐sensitized versus tick‐tick‐naive GP.

Transcript name and link to Uniprot Mouse or Rat ortholog	NCBI Identifier and link	Description	Log(2) fold change	*p*‐value	Function	Refs.
**Interleukins and other immune signaling products**						
Il19	100715337	interleukin 19	6.702564	5.25E‐06	This cytokine is found to be preferentially expressed in monocytes. It can bind the IL20 receptor leading to the activation of transcription 3 (STAT3).	[49]
Fgf23	100717518	fibroblast growth factor 23	7.578738	0.000567	Involved in positive regulation of B cell proliferation	[50]
Osm	100724699	oncostatin M	4.402756	2.18E‐09	Oncostatin M (OSM) contributes to extracellular matrix remodeling, hematopoiesis, differentiation, inflammatory response and proliferation.	[51]
Il13	100720118	interleukin 13	5.68252	0.009365	This gene encodes an immunoregulatory cytokine produced primarily by activated Th2 cells. This cytokine is involved in several stages of B‐cell maturation and differentiation.	[52]
Cd28	100714966	CD28 molecule	3.558919	0.000983	Involved in immune response, T cell activation; T cell receptor signaling pathway; and in positive regulation of T cell proliferation;	[53]
Cd69	100727950	CD69 molecule	3.525725	5.66E‐06	Expression Cd69 is induced upon activation of T lymphocytes and may play a role in their proliferation. Furthermore, the protein may act to transmit signals in natural killer cells and platelets.	[54]
Il24	100714873	interleukin 24	3.523712	0.002487	IL‐24 induces rapid activation of Stat‐1 and Stat‐3 transcription factors.	[55]
Fcrlb	100715396	Fc receptor like B	3.512076	3.69E‐05	FCRL1‐5 could regulate different features of B‐cell evolution such as development, differentiation, activation, antibody secretion and isotype switching.	[56]
Ms4a7	100719485	membrane spanning 4‐domains A7	3.214635	0.000782	This family member is associated with mature cellular function in the monocytic lineage, and it may be a component of a receptor complex involved in signal transduction.	[57]
Cd38	106026535	ADP‐ribosyl cyclase	3.388717	0.001823	Involved in positive regulation of B cell proliferation.	[58, 59]
Cd38	100726537	CD38 molecule	3.087273	2.44E‐06	Involved in positive regulation of B cell proliferation	[58, 59]
G3V7I1	100735368	C‐C motif chemokine 4	3.383439	0.000379	Involved in the immune response.	[60]
Q5Y4N7	100727811	adhesion G protein‐coupled receptor E4P	4.339531	0.004216	Enables G protein‐coupled receptor activity.	[61]
Fut7	100717385	fucosyltransferase 7	3.82816	0.000981	Involved in fucosylation.	[62]
**Cytotoxic T cells product**						
Clec1b	100726289	C‐type lectin domain	3.592165	6.55E‐09	Natural killer cells express multiple calcium‐dependent lectin‐like receptors that either inhibit or activate cytotoxicity and cytokine secretion	[63]
GRAB	100736180	granzyme B	6.598226	2.23E‐06	Granzyme B (granzyme 2, cytotoxic T‐lymphocyte‐associated serine esterase 1)	[64]
GRAC	100719669	granzyme C	5.393386	0.000195	Granzyme C (granzyme 2, cytotoxic T‐lymphocyte‐associated serine esterase 1)	[65]
GRAB‐Like2	100720219	granzyme B‐like	4.93077	6.42E‐07	Granzyme B (granzyme 2,—cytotoxic T‐lymphocyte associated serine esterase 1)	[64]
**Chemotaxis**						
LOC100734903	100734903	C‐C motif chemokine 3‐like	3.424106	0.000368	Involved in eosinophil chemotaxis.	[66]
LOC100735187	100735187	C‐C motif chemokine 3‐like	3.132743	0.000621	Involved in eosinophil chemotaxis.	[66]
LOC100731901	100731901	alveolar macrophage chemotactic factor	3.996885	1.19E‐08	Involved in immune response; involved in chemotaxis; and in defense response.	[67]
LOC100732171	100732171	platelet basic protein‐like	5.654068	5.28E‐05	Involved in chemotaxis	[68]
**Proteases**						
Matrix metallopeptidases						
Mmp3	100729101	stromelysin‐1	3.872797	1.80E‐11	Involved in collagen catabolic process; and in extracellular matrix organization.	[69]
Mmp7	Mmp4	matrix metallopeptidase 7	4.247669	4.95E‐07	Involved in collagen catabolic process; and in extracellular matrix organization.	[70]
Mmp8	100724670	matrix metallo**pe**ptidase 8, neutrophil collagenase	3.120398	2.30E‐05	Involved in collagen catabolic process; and in extracellular matrix organization.	[71]
Mmp10	100727690	matrix metallopeptidase 10	4.925036	1.64E‐10	Involved in collagen catabolic process; and in extracellular matrix organization.	[72]
Mmp13	100731756	matrix metallopeptidase 13	3.183712	1.77E‐05	Involved in collagen catabolic process; involved in extracellular matrix organization.	[73]
Other peptidases						
Kelz	100729473	Kell metallo‐endopeptidase	4.564278	4.43E‐05	Hydrolyses bradykinin and neuropeptides.	[74, 75]
Prss29	100724332	Serine protease 29‐like	6.958842	1.79E‐05	Involved in proteolysis.	[76]
**Prostanoid catabolism**						
CP4FE	100733735	cytochrome P450 4F3‐like	5.74894	7.25E‐05	Cytochrome P450 4F3 (CYP4F3), originally identified as one of the leukotriene B4 ω‐hydroxylases, participate in the metabolism of various endobiotics, as well as some xenobiotics.	[77]
**Innate immunity**						
APOBEC‐1	100730186	APOBEC‐1‐like	5.978293	0.007912	Nuclear C to U RNA editing—many of these candidate RNAs are involved in cytokine signaling; anti‐viral.	[78]
Oasl	100734390	2'‐5'‐oligoadenylate synthetase like	3.94653	4.14E‐05	Involved in defense response to virus; involved in negative regulation of viral genome replication; involved in regulation of ribonuclease activity	[79]
NGAL	100722819	neutrophil gelatinase‐associated lipocalin‐like	6.307814	0.0051	Enables iron ion binding activity. Involved in innate immune response, positive regulation of cold‐induced thermogenesis; and siderophore transport.	[80]
Kunitz serine protease inhibitor						
Spint3	101787904	Protease inhibitors‐like, Kunitz type, 3	3.861328	3.76E‐05	Protease inhibition.	[81]

**FIGURE 7:**
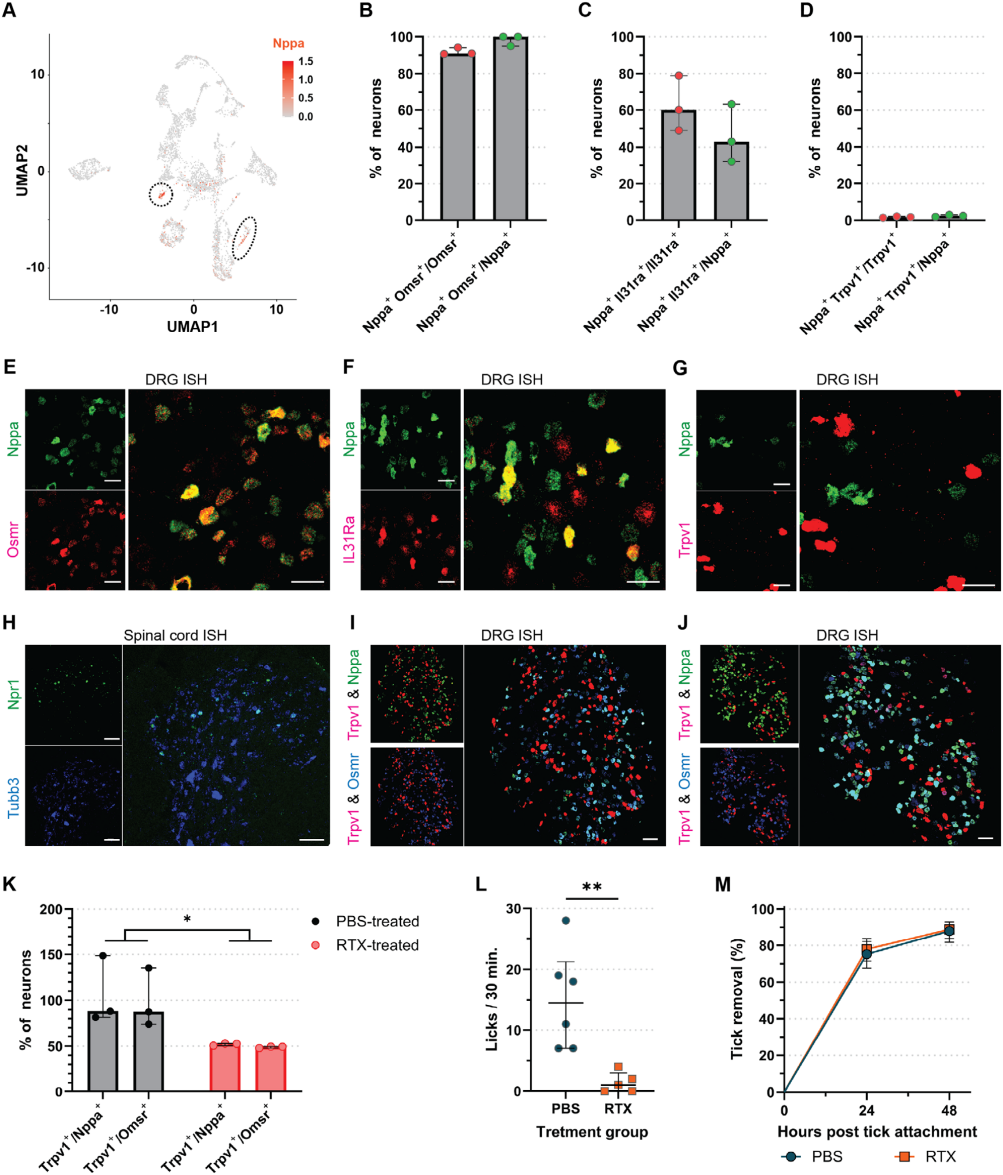
Immune‐induced itch in response to tick bite is not mediated through a Trpv1 neural pathway. (**A**) UMAP of scRNA‐seq of guinea pig (GP) dorsal root ganglia (DRG) neurons (GEO: GSE201654) highlighting Nppa expression. (B–D) Quantification of neurons co‐expressing Nppa and (B) Osmr, (C) IL31Ra, or (D) Trpv1 (*N*=3 GPs/staining). (B–D) Mann‐Whitney U test: (B) *p* = 0.1, (C) *p* = 0.4, (D) *p* = 0.2. Median ± IQR shown. (E–G) A representative multiplex ISH image showing co‐expression of (E) Osmr, (F) IL31Ra, or (G) Trpv1 on GP DRG Nppa^+^ neurons. Scale bar: 50 µm. (H) Expression of Npr1 on Tubb3^+^ GP spinal cord dorsal horn neurons. Scale bar: 50 µm. (I,J) A representative multiplex ISH image showing co‐expression of Trpv1, Nppa, and Osmr on GP DRG neurons in (I) PBS‐treated (*N* = 6) and (J) RTX‐treated (*N* = 5) GPs. Partial ablation of Trpv1 is observed in RTX‐treated GPs. Scale bar: 100 µm. (K) Quantification of partial Trpv1 ablation in DRG neurons by RTX‐treatment. Two‐way ANOVA: PBS‐ versus RTX‐treatment: *p* = 0.0235. Median ± IQR shown. (L) Number of licks to the capsaicin injection site in PBS‐ and RTX‐treated GPs over the course of 30 min. *N* = 6 GPs/group. Mann‐Whitney U test: *p* = 0.0022. Median ± IQR shown. (M) Tick removal on PBS‐ and RTX‐treated GPs (*N* = 6/group). Analysis by Peto & Peto modification of the Gehan‐Wilcoxon test: *p* = 0.5381. 95% CI is shown. ns > 0.05, ^*^ ≤0.05, ^**^ <0.01, ^***^ <0.001, ^****^ <0.0001. For detailed statistics see supplementary report.

Next, we wanted to explore whether the molecular neuronal signaling apparatus that triggers an itch response is present in GPs. For this, we examined single‐cell sequencing data from the dorsal root ganglia (DRG) of naive GPs from a previous study for molecules which might be involved in the detection of potential pruritogens from Table 1 [28]. Analysis of these data highlighted two transcriptionally distinct molecular populations of Nppa‐positive putative itch sensory neurons (Figure 7A) with enriched expression of the IL‐31 cytokine receptor complex for itch, Osmr and IL31Ra (Figures 7B,C) [23, 37]. Nppa is a close family homolog of Nppb and, like Nppb, activates the Npr1 receptor [38]. Single cell RNAseq data also indicated that these classes of neurons differed from similar murine and human itch neurons because of their lack of expression of Trpv1 as well as other itch specific genes (Figure 7D) [25, 26, 27]. To validate these unique expression patterns, we performed a multicolor label in situ hybridization (ISH). These studies confirmed that GP DRG contains a class of neurons expressing Nppa and Osmr (Figure 7E), IL31R (Figure 7F), but not Trpv1 (Figure 7G). In addition, we detected expression of the itch receptor Npr1 in neurons in the superficial lamina of the GP spinal cord (Figure 7H). Trpv1 is expressed on GP neurons not expressing Nppa (Figure 7G). Therefore, we hypothesized that ablation of Trpv1 afferent fibers should not affect the scratch response and consequently, tick removal. To test this hypothesis, we treated tick‐sensitized GPs with resiniferatoxin (RTX) a Trpv1 agonist which ablates Trpv1‐expresing neurons, or with control vehicle [39]. GPs treated with RTX displayed a large reduction of Trpv1‐expressing neurons relative to Nppa and Osmr‐neurons (Figure 7K), and they became insensitive to capsaicin, a Trpv1 agonist (Figure 7L). Importantly, RTX‐ treated GPs that are insensitive to capsaicin removed ticks as effectively as control‐treated GPs that are sensitive to capsaicin (Figure 7M). This indicates that tick removal by IITR is independent of the Trpv1 pathway. This contrasts with previous work showing that tick‐derived proteins induce itch in naive mice through the Trpv1 pathway [40]. Further experiments are needed to identify the neural pathway and the pruritogens responsible for IITR.

### 2.5 The Adaptive Immune Response That Leads to IITR Develops Within 5 Days After Initial Tick Attachment

**FIGURE 8:**
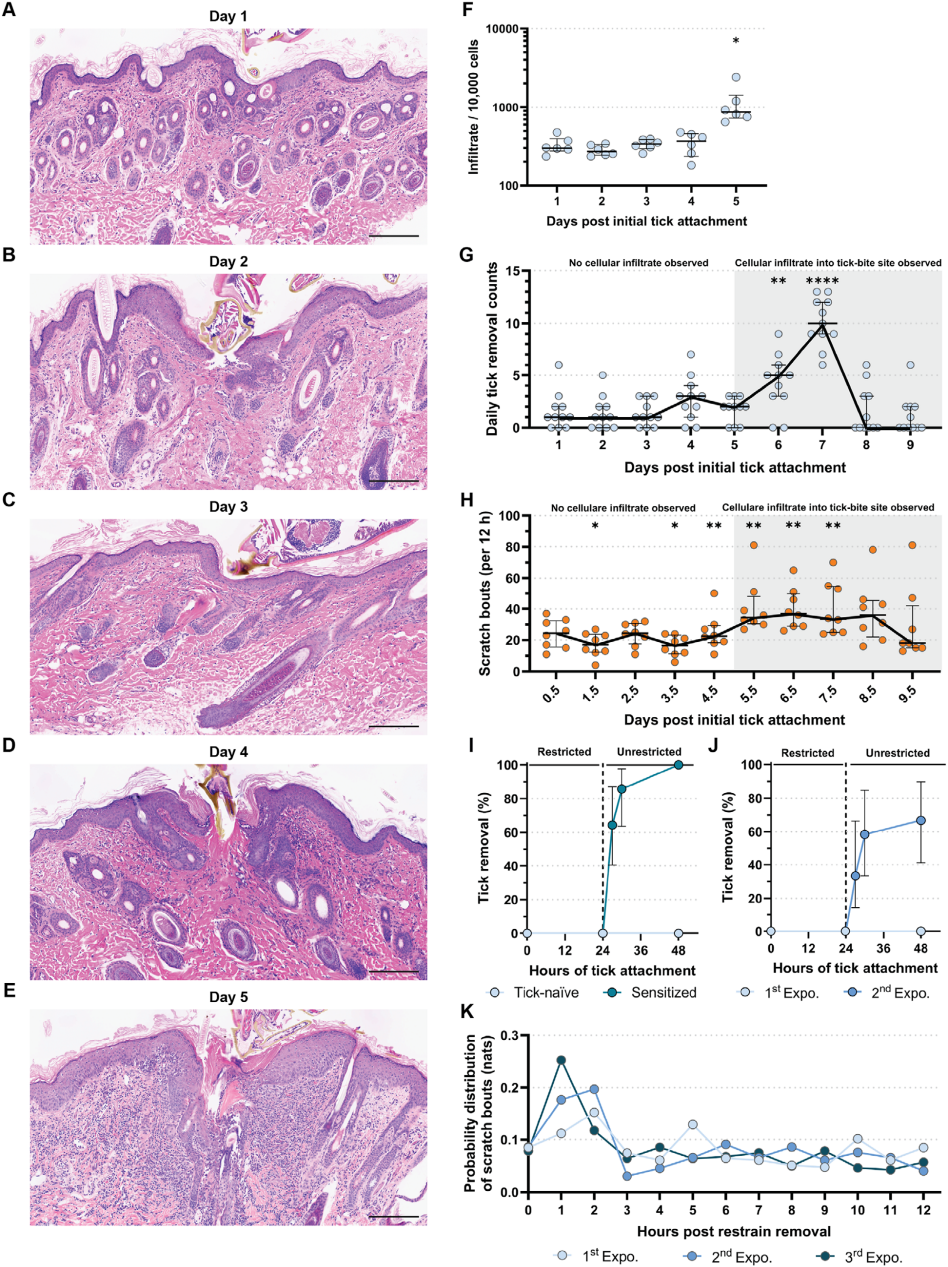
IITR is rapidly acquired and highly sensitive. (A–E) H&E skin cross‐sections (>|5µm|<) of tick‐bite sites at (A) 1 day, (B) 2 days, (C) 3 days, (D) 4 days, and (E) 5 days. Scale bar: 200 µm. (F) Cellular infiltrate quantification over days 1–5 post tick placement (pta). *N* = 6 sections/time‐point. Dunn's test: Day 1 versus Day 5: *p* = 0.023; Day 2 versus Day 5: *p* = 0.0016. Median ± IQR shown. (G,H) Tick‐naive guinea pigs (GPs) were continuously exposed to ticks over the course of 10 days. *N* = 8 GPs/time‐point. (G) Scatter plots showing daily counts of removed ticks. Pairwise estimated marginal means: 24 h pta versus 144 h pta: *p* = 0.0025; 24 h pta versus 168 h pta: *p* < 0.0001. Median ± IQR shown. (H) Scatter plots showing the total scratch bouts per 12 h recording over 10 days. Pairwise estimated marginal means: Day 1 versus day2: *p* = 0.0326; Day 1 versus Day 4: *p* = 0.0184; Day 1 versus Day 6; *p* = 0.0011; Day 1 versus Day 7: *p* = 0.0014; Day 1 versus Day 8: *p* = 0.0017; Day 1 versus Day 9: *p* = 0.0066. Median ± IQR shown. (I,J) Smoothed, inverted Kaplan–Meyer plot showing probability of tick removal (%) of a single tick at 27, 30, and 48 h pta after Elizabethan collar removal at 24 h pta. Showing 95% CI. (I) Challenge of tick‐naive (*N* = 12) and tick‐sensitized (*N* = 14) GPs. *N* = 15 ticks/GP. Analysis by Peto & Peto modification of the Gehan‐Wilcoxon test: *p* < 0.0001. (J) Tick sensitization with single ticks (*N* = 1 tick/GP/exposure): first (*N* = 12 GPs) and second (*N* = 12 GPs) tick exposures. Elizabethan collar removal at 24 h pta. Analysis by Peto & Peto modification of the Gehan–Wilcoxon test: *p* = 0.0008. (K) Probability distribution (normalization that allows direct comparison of distributions) of hourly scratch bouts measured in natural units of information (nats) against a single attached tick in a first, second, and third tick exposure. Data per exposure complied from *N* = 8 GPs. Pairwise two‐way Kolmogorov–Smirnov test: first versus second exposure: *p* = 0.9991; first versus third exposure: *p* = 0.9284; second versus third exposure: *p* = 0.9986. ns > 0.05, ^*^ ≤0.05, ^**^ <0.01, ^***^ <0.001, ^****^ <0.0001. For detailed statistics see supplementary report.

To investigate how fast the immune response leading to IITR develops, we exposed tick‐naive GPs to ticks and collected skin punch biopsies at days 1–5 post‐initial tick attachment (Figure 8A–E). On day 1 pta, a mild superficial dermal edema was observed (Figure 8A). On days 2–4, the epidermis at and adjacent to the site of the attached tick was 2–3 times the normal thickness with a minimal dermal inflammatory infiltrate at the tick bite site (Figure 8B–D). However, by day 5 pta, we observed a significant increase in dermal cell infiltrate surrounding and separating adnexal structures that extended to the overlying moderately hyperplastic epidermis (Figure 8E). Cell counts provided further evidence of a significant and sudden increase in cell infiltration at the tick‐bite site on day 5 pta (Figure 8F).

To investigate whether the observed sudden increase in cell infiltration 5 days pta corresponds to active tick removal by GPs, we implemented a continuous tick exposure protocol, replacing ticks every 3 days (Figure S3A) to circumvent natural loss of ticks that fed to repletion [41, 42]. This allowed us to observe whether increases in targeted scratch bouts and tick removal occur in conjunction with an increase in the local adaptive cellular immune infiltration. We counted the ticks daily and assessed how many were lost. We found that daily tick removal increased significantly on day 6 and peaked by day 7 pta (Figure 8G). Most ticks were removed by day 8 pta (Figure 8G; Figure S3B). The observed increase in the rate of tick removal was concurrent with a higher density of cellular infiltrate on day 5 (Figure 8E,F), suggesting an association between the cellular infiltrate and induction of IITR. In line with this observation, the increased rate of tick removal also corresponded to the onset of increased scratching at the tick bite site on day 5.5 pta that was maintained up to day 8.5 pta before subsiding on day 9.5 pta when most of the ticks had been removed (Figure 8G,H; Figure S3B). Collectively, these data show that the IITR‐associated neuroimmune response develops rapidly, within 5 days after a first exposure to tick bites, on GPs not previously exposed to tick bites, leading to an active itch response that results in IITR by 6 days post initial tick exposure.

### 2.6 Exposure to a Single Tick Bite is Sufficient to Generate IITR

**FIGURE 9:**
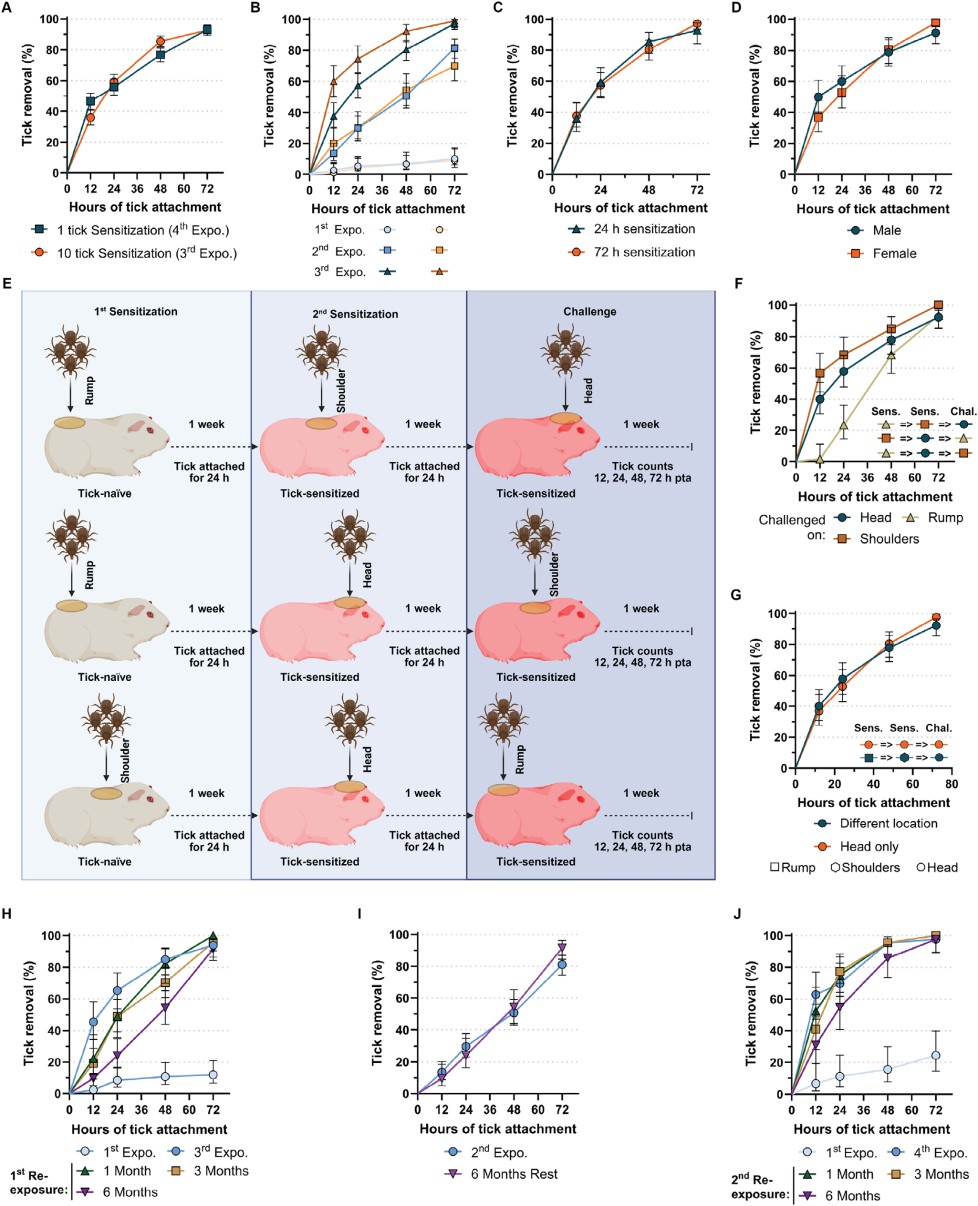
IITR is a very sensitive and robust anti‐tick response. (A–D) Smoothed, inverted Kaplan–Meyer plot showing probability of tick removal (%). Analysis by Peto & Peto modification of the Gehan–Wilcoxon test. 95% CI shown. (A) fourth and third weekly exposure of GPs sensitized with 1 and 10 ticks, respectively. *N* = 6 GPs/group; total *N* = 86 ticks (1 tick Sensitization), total *N* = 103 ticks (10 tick Sensitzation). *p* = 0.782. (B–D) GPs were exposed to 15 ticks per exposure. (B) Tick removal success in three times sensitized GPs exposed weekly (blue shades; *N* = 9 GPs/tick exposure) or every 3 weeks (orange shades; *N* = 6 GPs/tick exposure). Comparing first Exposures (Expo.): *p* = 0.8787; second Expo.: *p* = 0.8787; third Expo.: *p* = 0.0006. (C,D) GPs were sensitized weekly. (C) Third exposure of GPs sensitized over 24 h or 72 h per exposure (*N* = 6 GPs/group). *p* = 0.8484. (D) Third exposure of male and female GPs (*N* = 6 GPs/group). *p* = 0.3898. (E) Schematic of guinea pig (GP) sensitization and challenge depicting tick placement on different body locations (Created in BioRender: https://BioRender.com/y2smy29). (F,G) Smoothed, inverted Kaplan–Meyer plot showing probability of tick removal (%) in GPs sensitized weekly with 15 ticks. Analysis by Peto & Peto modification of the Gehan–Wilcoxon test. 95% CI is shown. (F) Shows results of tick challenge on different body locations on *N* = 6 GPs sensitized on other body locations than the challenge (see (E)). Head versus Rump: *p* < 0.0001, Shoulders versus Rump: *p* < 0.0001, Head versus Shoulders: *p* = 0.0483. (G) Tick removal success during a third exposure on the head in GPs sensitized either on the same or different locations (*N* = 6 GPs/group). *p* = 0.9403. (H–J) Smoothed, inverted Kaplan–Meyer plot showing probability of tick removal (%). Analysis by Peto & Peto modification of the Gehan–Wilcoxon test. 95% CI is shown. (H–J) Guinea pigs (GPs) were exposed to 15 ticks. (H) Tick removal success in GPs after 1, 3, and 6 months of rest (no tick exposure) compared to a first exposure of tick‐naive GPs or a third exposure of weekly sensitized GPs (*N* = 6 GPs/group). Third exposure (expo.) versus 1 Month [rest] (*p* = 0.0536) / versus 3 Months [rest] (*p* = 0.0025) / versus 6 Months [rest] (*p* < 0.0001). (I) Tick removal success of GPs during a second weekly exposure (*N* = 9 GPs) and after 6 months (*N* = 6 GPs) of no tick encounter. *p* = 0.6281. (J) Tick removal success of the same GPs as in panel (H) during a subsequent tick‐exposure a week later showing enhancement of tick removal success after long breaks from tick exposure (*N* = 6 GPs/group). Fourth exposure (expo.) versus 1 Month [rest] (*p* = 0.6205) / versus 3 Months [rest] (*p* = 0.3319) / versus 6 Months [rest] (*p* = 0.03). ns > 0.05, ^*^ ≤0.05, ^**^ <0.01, ^***^ <0.001, ^****^ <0.0001. For more details see supplementary report.

Next, we sought to determine whether a single tick would elicit IITR in tick‐sensitized GPs. We found that single ticks were removed by tick‐sensitized GPs within 3–6 h after collar removal, while tick‐naive GPs failed to remove ticks (Figure 8I). Remarkably, prior exposure to a single tick was sufficient to acquire the IITR response, efficiently triggering it during a subsequent challenge with one tick (Figure 8J). This provides evidence that a single tick can prime and sustain effective IITR. In fact, weekly exposures to a single tick over 3 weeks were as effective in sensitizing GPs as two exposures to ten ticks (Figure 9A). Importantly, scratch bout frequencies to one tick peaked within the first hours in GPs exposed twice or three times to single ticks and swiftly returned to baseline after tick removal (Figure 8K), supporting the hypothesis that early tick removal responses are mediated by IITR.

Of note, IITR responses were consistent regardless of whether there was a 1‐ or 3‐week gap between tick exposures (Figure 9B), whether ticks fed for 24 or 72 h (Figure 9C), or whether male or female GPs were used (Figure 9D). Together, these findings indicate that the elicitation of IITR is highly efficient.

### 2.7 The Immune Response Leading to IITR is Systemic and Long‐Lasting

Next, we investigated whether the immune response leading to IITR is a local or systemic response. GPs were exposed three times to 15 ticks at a specific body location, and IITR was measured at the third exposure to determine whether the site of tick bites influences its development (Figure 9E). Supporting a systemic response, tick removal efficiency was comparable regardless of the sensitization site (Figure 9F,G). Importantly, there was a significant increase in cell recruitment at tick‐bitten sites after tick re‐exposure on tick‐sensitized guinea pigs compared to non‐bitten sites on tick‐sensitized guinea pigs (Figure S4), supporting a systemic response.

Importantly, IITR remained intact for at least 6 months after a tick exposure event after one or two tick exposures (Figures 9, H and I), albeit with a slightly attenuated magnitude in the former (Figure 9H). Pertinently, IITR intensity was restored after a new tick exposure, even after a 6‐month‐long latency (Figure 9J). Cumulatively, our data strongly suggest that the immune response leading to IITR is long‐lasting and that IITR is a systemic defense mechanism, allowing a sensitized host to rapidly detect and remove attached ticks.

## 3 Conclusion

Our findings strongly support that an adaptive cellular immune response in conjunction with activation of neuronal itch pathways are responsible for IITR, an overlooked mechanism observed in tick‐sensitized GPs. Itch as a mechanism of tick loss has been proposed previously but never demonstrated or tested [15, 43]. This work provides a formal demonstration that an immune‐induced itch is responsible for an early and active tick removal by tick‐sensitized GPs that is independent of IgE or IgG antibodies. The unchanged number of mast cells in the skin of tick‐naive or tick‐sensitized GPs suggests that they are also not involved in this itch response. Specifically, IITR is established well before humoral responses develop, with initial tick sensitization of tick‐naive animals taking as little as 5 days. This observed adaptive period, albeit short, indicates that early tick removal is not initiated by innate immune responses alone. Instead, our results suggest that IITR‐mediated early tick removal relies on an adaptive immune response driven by the recruitment of T cells and macrophages, similar to a Type IV hypersensitivity response, followed by the triggering of a TRPV1‐independent itch pathway that leads to localized scratching. It is worth noting that IITR does not exclude the development of ATR and the involvement of IgE‐ and IgG‐activated innate cells at later time periods [43]. In fact, IITR as described here, differs from the previously described ATR in its methodology, kinetics, type of immune response, and effect on tick fitness. ATR experiments used ticks contained in “pill boxes” or “capsules” or used Elizabethan collars [7, 11, 44]. These experimental designs inadvertently prevented accessibility to the site where the animals are feeling the itch and hence precluded mechanical tick removal. ATR, unlike IITR, is dependent on antibodies, basophils, and mast cells, and compromises tick feeding, tick attachment, and reduces tick weight and egg laying [43, 45]. These temporal and mechanistic differences clearly distinguish IITR from ATR and demonstrate the occurrence of two distinct mechanisms that lead to different anti‐ectoparasite responses: an early acquired cell‐mediated immunity discovered here that drives rapid itching and active removal of ticks within 3–6 h, and a later antibody‐mediated basophilic immune response that primarily affects tick fitness [43, 45].

Others have shown that itch responses to mosquito bites develop only after repeated mosquito exposures [46]. Hypersensitivity and itch responses to mosquito bites were associated to high levels of IgE and IgG against mosquito salivary gland proteins in humans and were associated to IgE and IgG1 antibodies in mice, independently of mast cell degranulation or histamine [47, 48]. These responses are distinct from IITR, an antibody‐independent earlier immune response to tick bites that induces an itch response.

The rapid removal of ticks by tick‐sensitized animals in as little as 3–6 h is due to the neuroimmune response described in this work. The activation of neural itch pathways, most likely induced by the observed cell infiltrate, causes the tick‐sensitized animal to feel and remove ticks rapidly, in a window of time at or earlier than 24 h, before infected ticks transmit several major pathogens, including *Borrelia burgdorferi* [5]. This window of time may be vital for the development of effective therapeutic strategies for prevention or reduction in the occurrence of ectoparasite‐borne diseases such as Lyme disease, rickettsioses, ehrlichiosis, anaplasmosis, and other infections transmitted by *Ixodes scapularis*. These innovative strategies are based on triggering the body's own itch‐based defense systems. In one study, subjects reporting itch in association with tick bites had a lower probability of acquiring Lyme disease [15]. Based on our findings, we suggest that these individuals probably removed the attached ticks before pathogen injection.

Here, we show that GPs naturally induce a neuroimmune response to tick bites that leads to a scratching response and consequently tick removal. In contrast, mice, including natural reservoirs or strains used in preclinical models, generate a skin immune response to tick bites but without active tick removal [46]. Then it appears that tick‐sensitized mice do not scratch in response to tick bites and do not mechanically remove ticks even in the presence of a skin immune response [46]. This indicates that the active neurological component that triggers IITR and the scratching response to tick bites may be absent in mice due to their distinct neuroimmune circuits. The mouse model is therefore ideal for the study of ATR, characterized by a basophilic response and anti‐tick antibodies that directly affect tick fitness [47]. Alternatively, we propose GPs as an inducible model to study itch responses to blood‐feeding arthropods, providing an opportunity to investigate the mechanisms responsible for ectoparasite sensing and removal.

A short time, 5 days from initial tick exposure, is required to develop the immune response that leads to IITR. This suggests that an intervention based on itch‐induction may be effective in preventing pathogen transmission shortly after immunization. This is in contrast to ATR, which has been shown to require between 60 to 90 days after initial tick exposure for its development [14]. Importantly, our demonstration that a single tick can prime the development of this fast neuroimmune response to tick bites suggests that a small dose of itch‐inducing antigen/s will be sufficient for an effective anti‐tick vaccine. The rapid subsiding of itch post tick removal is another advantage for a vaccine as it precludes unnecessary discomfort, or even damage to the host skin.

Our study provides evidence that itch serves as a defensive mechanism for the removal of ectoparasites, proposing a potential basis for the evolutionary conservation of itch. It will be important to determine whether similar adaptive responses are employed for defense against other ectoparasites. Although we hypothesize that ectoparasite removal is the major evolutionary drive for the development of itch, our results do not exclude the contribution by other potential mediators such as inflammation [48]. Similarly, our results do not exclude other non‐itch‐mediated processes being involved in defense against ectoparasites.

In summary, we provide evidence for the presence of IITR. Driven by an early neuroimmune response to tick bites in a tick‐sensitized host, IITR causes localized and temporary itching and a fast mechanical tick removal and occurs in a window of time that precedes transmission of many tick‐borne pathogens. Further understanding of this neuroimmune response may lead to the identification of itch‐inducing tick antigens that can be developed as an innovative “behavioral assisted” anti‐tick vaccine.

## 4 Limitations of the Study

Although the guinea pig is the most appropriate model for studying immune‐induced tick removal, it has important limitations, including the lack of immunological reagents needed for the characterization of host immune responses using e.g., flow cytometry, histology, and other antibody‐based assays. In addition, guinea pigs are not an animal model with well‐developed genetic strains available to dissect the neural components of the itch response. Translating our findings from guinea pigs to humans presents inherent challenges. Guinea pigs differ from humans in several aspects of skin structure, innate and adaptive immune signaling, and inflammatory cell recruitment, all of which can influence how ticks attach, feed, and are rejected by the host. Guinea pig's patterns of antibody responses, cytokine profiles, and hypersensitivity reactions to tick saliva may not fully mirror those observed in humans, limiting the extent to which our mechanistic conclusions can be directly applied to human tick–host interactions.

## 5 Experimental Section

### 5.1 Ethics Statement

All experimental animal procedures were reviewed and approved by the National Institute of Allergy and Infectious Diseases (NIAID) Animal Care and Use Committee (ACUC) under animal protocol ASP LMVR6. The NIAID DIR Animal Care and Use Program complies with the Guide for the Care and Use of Laboratory Animals and with the NIH Office of Animal Care and Use and Animal Research Advisory Committee guidelines. Detailed NIH Animal Research Guidelines can be accessed at https://oma1.od.nih.gov/manualchapters/intramural/3040‐2/. Efforts were made in the planning and execution of all animal procedures to comply with the guidelines of the National Center for Replacement, Refinement, and Reduction (3Rs) of Animals in Research (https://nc3rs.org.uk/the‐3rs).

Human skin samples were obtained from participants enrolled in protocol NCT05036707. The study is approved by the institutional review board (IRB) at the National Institutes of Health (NIH) (Bethesda, MD). All participants signed written informed consent.

### 5.2 Guinea Pigs

3–6 weeks old out‐bred female or male albino Hartley guinea pigs, *Cavia porcellus* (Stain code: 051, https://www.criver.com/products‐services/find‐model/hartley‐guinea‐pig?region=3611), were obtained from Charles Rivers laboratories, Wilmington, MA, USA, and were communally housed in groups of up to three animals under static cage conditions at the NIAID Twinbrook animal facility, Rockville, MD.

### 5.3 Ticks

Pathogen‐free *Ixodes scapularis* nymphal ticks were either obtained from the Centralized Tick Rearing Facility, Department of Entomology and Plant Pathology, Oklahoma State University, Stillwater, OK, USA, (main source) or sourced from the established *Ixodes scapularis* colonies at the Rocky Mountain Laboratories (RML), NIAID, Hamilton, MT, USA (secondary source) and the Laboratory for Malaria and Vector Research (LMVR), NIAID, NIH, Rockville, MD, USA (temporary source). Nymphal ticks were housed in cotton‐wool‐sealed plastic tubes or gauze‐mesh‐sealed universal tubes, both sealed in zip‐lock bags containing a moist sponge for humidity. These bags were kept for long‐term storage in a housing incubator at 20°C and ≥ 95% humidity under a 16:8 h photoperiod at the Laboratory for Malaria and Vector Research (LMVR), NIAID, NIH, Rockville, MD, USA.

### 5.4 Experimental Guinea Pig Exposures to Nymphal Ticks

For experimental use, nymphal ticks were transferred from the cool housing incubator to a warm (∼27°C / 95%–98% humidity) incubator up to 4 days prior to experimental use. To break diapause and for acclimatization, zip‐lock bags containing nymphal ticks were transferred to a windowsill for sunlight exposure at room‐temperature 1–2 days prior to experimental use and left there until use. For nymphal tick exposures, the head and neck area of GPs were shaven (not too closely) with electrical clippers, leaving 1–2 mm stubble. Our nymphal ticks preferred the stubble over depilated skin as it facilitated holding on to the host and stimulated their sensilla once they burrowed in between the stubble. GPs were then anesthetized individually in 5–7 min intervals by intraperitoneal (i.p.) administration of a Ketamine (50 mg kg^−1^)/Xylazine (5 mg kg^−1^) mixture according to body weight. Using a fine paint brush, nymphal ticks were stimulated to burrow in between the remaining hair stubbles by repeatedly tapping them lightly in intervals during the first 10–15 min post placement. By brushing hard against the grain of the hair stubble, nymphal ticks that would not attach to the host skin were identified and replaced with new nymphal ticks. This cycle was repeated until the desired number of ticks had attached. This procedure increased the likelihood of tick attachment at a target location to > 95%. GPs were placed for recovery (60–90 min post‐induction) in triple containment cages, where the outermost cage contained a water barrier and rims coated with Vaseline to prevent tick escape. ∼3 h post tick attachment (pta), GPs were anesthetized with isoflurane (Forane by Baxter, Deerfield, IL, USA) gas (4% at 2 L min^−1^ O_2_ flow rate), and attached nymphal ticks were counted. If applicable, attached nymphal tick numbers were reduced to a desired number for all GPs in an experiment. GPs were also checked for nymphal ticks that had bitten off‐target, which were removed, too. In some experimental tick exposures, Elizabethan collars (e‐collars; Lomir Biomedical Inc., Malone, NY, USA) were placed around the GPs’ necks to prevent premature tick removal by the GPs and approximately equal antigen delivery. Tick‐exposed animals were also housed individually to prevent social grooming. At the end of experimental tick exposures, remaining nymphal ticks were removed with tweezers, and GPs were returned to communal housing cages.

The maximum permitted duration of nymphal tick attachment during a nymphal tick exposure varied between experiments. In general, GPs were sensitized by weekly nymphal tick exposures lasting 24 to 72 h each as required and resting periods of 6 and 4 days, respectively. Only in one experiment, 18 days of rest were observed between exposures. Depending on the experiment, remaining nymphal ticks were counted at 12 h pta, 24 h pta, 36 h, 48 h pta, and/or 72 h pta

### 5.5 Tick Health Assessment

Tick‐naive and three times sensitized GPs were exposed to ticks as described above, and e‐collars were applied at 3 h pta after tick adjustments to 15 attached nymphal ticks. At 48 and 72 h pta attached nymphal ticks were recovered from half of the exposed GPs in each group, respectively. Care was taken to remove nymphal ticks by pulling gently with fine forceps to leave the hypostome intact. Recovered nymphal ticks were immobilized on double‐sided tape and imaged under a stereomicroscope (Leica M165 FC) with a Leica DFC 7000 T camera (Leica, IL, USA) using the Leica Application Suite X v.3.7.4.23463, which was also used for measurements of the length of the nymphal alloscutum's distention and the scutum's width (Figure 2D). These measurements were used to calculate the scutal index, which is a ratio produced by dividing the alloscutum's length by the scutum's width [82]. The scutal index is an established indicator of tick feeding success, in particular, in instances when ticks are not allowed to feed to repletion or non‐adult tick stages (here, nymphs) are used, excluding assessment of tick molting or egg mass. The concept is that length measurement of the alloscutum will increase with continued feeding over time, while the scutum remains unchanged during the feeding and thus, its width is fixed. Thus, the larger the ratio, the greater the engorgement. Further, nymphal ticks were visually assessed for intactness and movement under the stereomicroscope as indicators of nymphal tick life‐status; visible intactness and movement = alive, no movement despite stimulation by tapping with a fine paint brush or visible damage to/desiccation of the tick = dead.

### 5.6 Scratch Bout Quantification

We employed PhenoTyper home cages with video tracking technology using EthoVision XT v.17 for video analysis (Noldus Information Technology INC, VA, USA) to assess changes in guinea pig behavior post tick attachment. We employed an infrared camera system to record videos overnight in total darkness. GPs are often described as crepuscular, but studies have shown that their sleep occurs in short bursts and is evenly distributed over 24 h [83]. As animals were recorded for 14 h, we provided custom‐made absorbent cage liners (Top Layer: 85 gsm brushed/knitted polyester; Middle Layer: 250 gsm Polyester Soaker; Bottom Layer: 85 gsm brushed/knitted polyester bottom with waterproof TPU finished top) that were dyed with an infrared absorbent dye, which gave the liners a black appearance under the infrared camera. Experimentally, we exposed GPs to nymphal ticks as described above and either recovered them from the ketamine/xylazine general anesthesia in the PhentoTyper home cages after tick placement or moved them into the PhentoTyper home cages ∼5 h after nymphal tick placement. Black curtains were drawn around the cage to reduce stray room light and obstruct guinea pig vision of the room to avoid behavioral freezing. In EthoVisionXT, video tracking was conducted post‐recording and commenced when, first, the center point of a guinea pig was detected for ≥ 5 s, and, second, the GPs’ activity exceeded 0.05%, which indicated presence in the cage or recovery from anesthesia. Video tracking covered the first 12 h post‐anesthesia recovery or presence in the cage depending on experiment. Scratch bouts to the top of the head were used as an indicator of nymphal tick‐induced itch, according to the definition established by Shimada & LaMotte (2008) [84]. For the video analysis we defined potential scratch bouts in EthoVision XT by combining two “multi condition” criterions, consisting of the following criteria: (a) “body elongation” (threshold: <47%, Average: 10), “activity” (≥ 0.1%, Average: 10), and (b) “body elongation” (threshold: <46.5%, Average: 10), “distance moved” (nose‐point: <0.58 cm; center‐point: <0.3 cm; Track Smoothing (Lowess): 9), “activity” (≥ 0.26%, Average: 10), “velocity” (nose‐point: <12 cm/s; center‐point: <5 cm/s; Average: 10), “in zone” (Body points: nose point; not in the following zone: feeder zone). The output data was screened in R statistical software to narrow possible scratching events by excluding all events shorter than (a) 1 s, and (b) 0.66 s, respectively. Further, all events that showed an overlap of < 20% of a) and b) were excluded. All filter events were manually verified and scored in EthoVision XT and used as the basis to quantify scratch bouts (duration and frequency) for the statistical analysis.

### 5.7 Enzyme‐Linked Immunosorbent Assay (ELISA)

Detection of anti‐*Ixodes scapularis* IgG and IgE in tick‐naive and weekly exposed GPs for three (animals from week 1, 2, 3, and 4 groups) or four times (animals from week 10 group), was analyzed by indirect ELISA IgG using *I. scapularis* salivary gland homogenate (SGH), and a commercial IgE kit (MyBioSource, Inc., San Diego, CA, USA). Briefly, for IgG assay, microtiter plates were coated with 5 ug mL^−1^ of SGH and incubated overnight at 4°C. Subsequently, plates were blocked with per 10% FBS in PBS during 1 h at room‐temperature. Plates were washed six times with 0.05% Tween‐20 in PBS and incubated with serum diluted 1:500 in 5% FBS in PBS in duplicate. After 1 h at room‐temperature, plates were washed six times, and wells were filled with the secondary detection antibody (Goat anti‐Guinea Pig IgG (H+L)—Invitrogen—diluted to 1:5000 in 5% FBS in PBS). The plates were incubated for 1 h at room‐temperature. After twelve washes, the one‐Step ABTS substrate solution was added. The optical density (OD) was measured at 405 nm, using a 96‐well microplate reader after 30 min. An ELISA IgE assay was performed following the manufacturer's recommendation. Briefly, guinea pig sera were diluted 1:16, and spiked‐in samples were used as a control (final concentration 50 ng mL^−1^). After 15 min, the HRP reaction was stopped, and the OD was measured at 450 nm. The IgE concentration was assessed using a standard curve, and the interpolated values were obtained using a linear regression calculated with GraphPad Prism software.

### 5.8 Disruption of Establishment of Acquired Tick Removal

Fingolimod (FTY720) is an immunomodulator drug that modulates the sphingosine 1‐phosphate (S1P) receptors on naive lymphocytes, preventing their egress from lymph nodes [35, 85]. Five mg of FTY720 (Sigma–Aldrich) were suspended in 3 mL of PBS (Lonza. Portsmouth, NH) under sterile conditions by pipetting the suspension up and down several times. The suspension was transferred to a 15 mL Falcon tube and cycled three times through 10 min heating in a water bath at 37°C followed by vortexing for 10 min at 2000 rpm at room‐temperature. Tick‐naive GPs were treated with 4 mg/kg of the FTY720 suspension starting 5–7 days prior to the first exposure. Circulating lymphocytes were counted pre‐treatment and the day before exposure to assess FTY720‐induced reduction in circulating lymphocytes. Treatment continued until the end of the experiment. Nymphal tick exposures were conducted as described above.

### 5.9 Resiniferatoxin Administration

Resiniferatoxin (RTX) (1 mg) was obtained from AdipoGen Life Sciences (CA, USA). RTX was solubilized in 200 µL 100% ethanol to make a RTX stock concentration of 5 µg µL^−1^. It was further diluted to a working concentration of 100 ng µL^−1^ RTX in sterile vehicle (0.25% Tween 80, 2 mm ascorbic acid, 0.9% NaCl). Intrathecal injections (2 mg RTX in 20 µL working solution) in the guinea pig were made using a 500 µL zero dead volume insulin syringe with a fixed needle of 29G (MHC Medical Products, OH, USA). To test for loss of Trpv1‐neuron activity, intraplantar administration of 20 ug Capsaicin (Cat#M2028, Millipore Sigma, USA) in 20 µL of vehicle solution (5% Tween 80, 5% Ethanol, 0.9% NaCl) was made 7 days after RTX injection and number of licking bouts counted.

### 5.10 Transcriptome Sequencing and Analysis

Total RNA was extracted from skin biopsies as previously described [86]. Briefly, biopsies were stored in DNA/RNA Shield (Zymo Research) and kept at 4°C until processing. Skin was homogenized using 3 mm zirconium beads (Biolink Laboratories) and a Magnalyser bead beater (Roche Diagnostics). After homogenization, total RNA was loaded at InnuPure C16 touch (AnalitikJena) for magnetic separation of total RNA using an InnuPREP RNA Kit–IPC16 (AnalitikJena). Total RNA was sent for library preparation and sequencing (Novogene Co.). mRNA was enriched by Poly (A) capture and RNA libraries were prepared using the NEBNext Ultra II Directional RNA Library Prep Kit for Illumina (New England Biolabs) and sequenced on a NovaSeq 6000 (Illumina), generating paired‐end reads at 150 bp length. Raw RNA sequences were prepared and analyzed using the CLC genomics workbench v22 (Qiagen). Sequences were trimmed (prepare raw data workflow) to remove poor quality sequences and adaptors. Trimmed reads were mapped, and the differential expression of genes for the groups (three times sensitized Tick vs one‐time sensitized vs unbitten skin) was calculated for 4 biologically independent samples (RNA‐seq and differential gene expression analysis workflow). The NCBI Cavpor 3.0 assembly and annotation were used as a reference genome. As previously described, filtering was performed on all mapped gene counts to exclude genes where the sum of counts in all conditions was inferior to 10 [87]. Significant associations were considered when a *p* <0.01 and log_2_ fold change larger than 1.5 (+/‐). The principal component analysis (PCA) was performed with log_2_ TPMs +1. Heatmap was generated with log_2_ TPMs +1 z‐scores. Heatmap and PCA plots were generated using the GraphBio web app68.

### 5.11 Laser Speckle

Laser speckle contrast imaging is a non‐invasive technology that detects the dynamic change in backscattered light (cellular motion increases light scatter) [88]. It is used in clinical settings to assess perfusion of various tissues, which includes cellular influx into sites of skin inflammation. GPs were tick‐exposed as described above. 24 h pta, GPs were anesthetized by isoflurane delivered by a SomnoFlo vaporizer (Kent Scientific, Torrington, CT, USA), and hair around tick‐bite sites (top of the head) was removed by Nair (Church & Dwight Co., Inc., Ewing, NJ, USA) treatment. Briefly, Nair was applied according to supplier's instructions for 3 to 5 min (longer treatment times were detrimental to tick survival). Nair was washed off under running water. The following day, GPs were anesthetized individually by i.p. administration of a Ketamine (50 mg kg^−1^) / Xylazine (5 mg kg^−1^) mixture according to body weight and placed on a PhysioSuite heating pad (Kent Scientific, Torrington, CT, USA). Nymphal ticks attached to anesthetized GPs were visualized with a PerCam PSI HR (Perimed, NV, USA) calibrated using a LI723 CalBox PSI HR (Perimed) and the PIMsoft package v1.5 (Perimed). 1 cm^2^ skin areas were recorded for 1 min at a time. Circular regions of interest (ROI; 3.47 mm^2^) were drawn around each tick (excluding the tick itself, as it masked the signal) and one unbitten area per image for background signal measurements. Measurements were recorded for 1 min and subsequently averaged (smoothed) over a time of interest (TOI) of a 20 s range of the 1 min recoding. All readings were normalized by subtracting the background value from the measurements around the tick by the respective image, producing Δ perfusion units.

### 5.12 Histology

Skin biopsies of nymphal tick bitten, and unbitten skin (ø 3 mm) were collected by punch biopsy from CO_2_ euthanized GPs. Fresh biopsies were immediately placed in 10% neutral formalin and incubated 24 h at 4°C for thorough tissue fixation. Fixed skin biopsies were washed in PBS, stored in 70% ethanol, and submitted to Histoserv, Inc. (Histoserv, Inc., MD, USA) for paraffin embedding, sectioning, and staining with hematoxylin and eosin (H & E; provided by Histoserv). All paraffin skin sections were cut at 5 µm thickness. Samples were collected from tick‐naive, immunosuppressed (FTY720), and tick‐sensitized GPs. Stained slides were digitalized using a Motic Easyscan Pro 24 slide scanner. H&E images were processed and quantified for cellular infiltrate using Qupath software v0.5.1 [89]. The cell detection workflow was applied to IHC images. Machine learning object classification by random trees was applied on skin biopsies scans with at least 20 points per category. The cell detection workflow was applied to all images after algorithm training.

### 5.13 Immunohistochemistry

Formalin‐fixed paraffin‐embedded (FFPE) tissue sections (5 µm thickness) obtained from guinea pig skin were used to perform immunohistochemical (IHC) staining using the following antibodies: a rat monoclonal CD3 with colon CD3‐12 (AbD Serotec, Catalog No: MCA1477, dilution of 1:600) and a rabbit polyclonal Iba1 (Wako, Catalog No: 019‐19741, dilution of 1:800). Staining was carried out on the Bond RX (Leica Biosystems) platform according to manufacturer‐supplied protocols. Briefly, 5 µm sections were deparaffinized and rehydrated. Heat‐induced epitope retrieval (HIER) was performed using Epitope Retrieval Solution 1, pH 6.0, heated to 100°C for 20 min. The specimen was then incubated with hydrogen peroxide to quench endogenous peroxidase activity prior to applying the primary antibody. Detection with DAB chromogen was completed using the Bond Polymer Refine Detection kit (Leica Biosystems, Catalog # DS9800). Slides were finally cleared through gradient alcohol and xylene washes prior to mounting and placing cover slips. Sections were examined by a board‐certified veterinary pathologist (DAA). Immunohistochemistry images were processed and quantified using Qupath software v0.5.1 [89]. For macrophage staining (Iba1), ‘Cytoplasm: DAB OD mean’ was the score compartment, with a threshold of 0.05 for intensity parameters. For T‐cell staining (CD3), ‘Nucleus: DAB OD mean’ was the detection setting and the threshold was 0.1 for intensity parameters. All other settings were left as default. ROIs were determined based on the area of highest infiltration for the largest biopsy analyzed for each figure.

### 5.14 In Situ Hybridization and Imaging

Fresh frozen GP DRGs were sectioned at 20 µm with a cryostat. Multiplex in situ hybridization was performed with RNAscope multiplex fluorescence assay (Advanced Cell Diagnostics, Inc). Guinea pig spinal cords or dorsal root ganglia were embedded into cryostat embedding media, freshly frozen, and then cryosectioned at 20 µm thickness. RNAscope probes for Nppa (REF#1555751‐C1), Tubb3 (REF#1556171‐C2), Trpv1 (REF#1231081‐C3), Npr1 (REF#1574901‐C1), Il31ra (REF#1574911‐C2) and Osmr (REF#1231091‐C1) were included in this study. Fluorescence images were collected with a Nikon Eclipse Ti confocal laser‐scanning microscope.

### 5.15 Statistical Methodologies

#### 5.15.1 Software Packages

All statistics were conducted in RStudio v.2024.04.1‐748 using R v.4.4.3 with the exception of bulk RNAseq analysis in Figure 6 (details in Transcriptome sequencing and analysis section) and the neurological itch marker expression data in Figure 7, Figures S2 and S4, which were analyzed in GraphPad Prism v. 10.2.3 [90, 91]. All R packages employed in the data analyses are stated within the supplementary statistics report.

All data charts presented in the figures were generated in GraphPad PRISM v. 10. 2.3 with the exception of Figures 6A,B, and 7A, which were generated in Adobe Illustrator v. 29.4 (64‐bit; https://www.adobe.com/products/illustrator.html) along with the two tables in Figure 2A,B [92]. Experimental schematics were generated in BioRender (Figure 1A: https://BioRender.com/47zaovj; Figure 1C: https://BioRender.com/2jxmm9k; Figure 4A: https://BioRender.com/rduvqd2; Figure 9E: https://BioRender.com/y2smy29; Figure S3A: https://BioRender.com/dtivqaj).

#### 5.15.2 Statement on Experimental Design

We worked solely with albino Hartley guinea pigs obtained from Charles River Laboratories (see above). We used only female guinea pigs, which made it easier to randomly assign guinea pigs to treatment groups, after confirming that our results did not differ in males. Animals were selected into treatment groups at random by a researcher, taking care that treatments were mixed in a communal holding cage to avoid litter bias, as these were outbred guinea pigs. The researcher who assigned animals to treatment groups did not reveal the assigned treatment until after data analysis to ensure blinding, where possible. Repeated tick exposures on the same guinea pig were an exception as there was no logical way to blind the researchers handling the guinea pigs from week to week, and it was clear from which week the data stemmed.

Sample sizes were in general not estimated prior to the first experimental replication as there was no previous data available on IITR. In general, the first experimental replication (occasionally, the first two experimental replications) was used for sample size estimation, where possible, and additional experimental replications were conducted to achieve estimated sample sizes. In general, two to four experimental replications were conducted per experiment, trying to limit the number of animals used to the essential minimum to avoid waste of life. In general, 6 guinea pigs were handled per experimental replicate as this constituted the number of animals that could the reliably handled for tick infestation at a time. In some cases, sample size calculation had to be done retrospectively to confirm that a minimum power of 80% was maintained. Sample *N* are stated for all experiments in the figure legends and can also be obtained from the supplementary statistical report.

Experimental design adjustments were made to adhere to the 3Rs. E.g., we settled for time‐to‐event data collection on tick removal that allowed the tick to be the central *N*. By placing multiple (mostly 15) ticks on each guinea pig, this drastically reduced the required number of guinea pigs by 15‐fold. The guinea pig was then integrated in a hierarchical analysis as a layer of potential data variability, while the statistical focus was on the tick.

#### 5.15.3 Statement on Statistical Analysis

There is a separate detailed supplementary statistics report provided with this study available through the journal's web page. Abridged information on sample *N*, applied statistical tests, *p*‐values and presented statistics in the charts can be found in the figure legends for each chart. In general, all tests were two‐tailed where applicable. Wherever parametric statistical tests were considered for data comparison, sample data was assessed for Gaussian distribution by Shapiro–Wilks test, QQ plots, and assessment of skewness and kurtosis. In cases of small sample *N*, it was considered that assessment of data distribution was unreliable and if deemed necessary, tests were selected that could adjust for assumption violations, e.g., robust two‐way ANOVA, instead of standard two‐way ANOVA. Homogeneity of variance was assessed by Levene's test, and where applicable, homogeneity of covariance was assessed by use of the Box's M‐test. In cases of two‐way or three‐way ANOVA, Mauchly's test for sphericity was automatically executed with the anova_test() function from the rstatix package, and the Greenhouse‐Geisser sphericity correction was automatically applied in cases of violation. We also checked for outliers employing the identify_outliers() function from the rstatix package. In cases of outcome variable correlation, the linearity assumption and, where applicable the multicoliniearity assumption were tested with the correlation test function cor_test() from the rstatix package. In the case of multicollinearity the r statistic needed to be within the range [0.9, ‐0.9]. Survival data were analyzed by both the Peto & Peto modification of the Gehan–Wilcoxon test and the Cox proportional hazards model. The latter was also used for the production of hazard ratios. It also allowed for the incorporation of hierarchical analysis, which mattered in some cases where the guinea pigs contributed significantly to data variability. Count data, like the scratch bout data, were analyzed by types of Poisson and negative binomial regression analysis, depending on assumption tests, while cross‐tabled data were either analyzed by Chi‐square or Fisher's exact test, depending on assumption tests. In cases of multiple pairwise comparisons, tests were chosen that inherently adjust p‐values for multiple comparisons, e.g., Tukey's test or Dunn's test. Wherever the authors had to select the adjustment method manually, the False‐Discovery Rate test, aka FDR or Benjamini‐Hochberg test, was selected. For comprehensibility, results section and figure legends contain only information on final outcomes and limit reporting *p*‐values of < 0.05, while everything else is reported in the supplementary statistics report.

## Funding

This research was supported by the Intramural Research Program of the National Institute of Allergy and Infectious Diseases (NIAID), grant numberAI000932, the National Institute of Dental and Craniofacial Research (NIDCR), the National Institutes of Health (NIH).

The Contribution of the NIH author(s) were made as part of their official duties as NIH federal employees, are in compliance with agency policy requirements, and are considered Works of the United States Government. However, the findings and conclusions presented in this paper are those of the author(s) and do not necessarily reflect the views of the NIH or the U.S. Department of Health and Human Services.

## Note

This article reflects the views of the author and should not be construed to represent FDA's views or policies. The content of this publication does not necessarily reflect the views or policies of the Department of Health and Human Services, nor does mention of trade names, commercial products, or organizations imply endorsement by the U.S. Goverment.

## Conflicts of Interest

The authors declare no conflict of interest.

## Supporting information




**Supporting File**: advs73764‐sup‐0001‐SuppMat.docx.

## Data Availability

All data are available in the manuscript or the supplementary materials. Materials described in this manuscript are available from J.G.V. (jvalenzuela@niaid.nih.gov) on request. All raw and processed sequencing data are available at NCBI, BioProject ID: PRJNA1259463, Reviewer link: https://dataview.ncbi.nlm.nih.gov/object/PRJNA1259463?reviewer=r47cm3ftc888ia8giu1s072obe. All data underlying the statistical analyses in the supplementary statistics report is also available from the author's GitHub repository: https://github.com/joedoehl/An‐Adaptive‐Neuroimmune‐Response‐Driving‐Itch‐and‐Fast‐Tick‐Removal.git. Data from the manuscript is currently in the following private link: https://figshare.com/s/cff69f6c54b0a86f5fef. All R scripts underlying the statistical data analysis and the raw data are available at https://github.com/joedoehl/An‐Adaptive‐Neuroimmune‐Response‐Driving‐Itch‐and‐Fast‐Tick‐Removal.git.
