# Supplementary material for: Discovery of an Adaptive Neuroimmune Response Driving Itch and Fast Tick Removal with Implications for Preventing Pathogen Transmission
Source: Adv Sci (Weinh). 2026 Jan 27;13(32):e17742. doi: 10.1002/advs.202517742 (PMC13252611; doi:10.1002/advs.202517742)
Supplement: Supplementary file 1 — Supporting File: advs73764‐sup‐0001‐SuppMat.docx. [file ADVS-13-e17742-s001.docx]

**Supporting Information**

Supporting Information is available from the Wiley Online Library or from the author.

Supporting Information

**Discovery of an Adaptive Neuroimmune Response Driving Itch and Fast Tick Removal with Implications for Preventing Pathogen Transmission**

Johannes S. P. Doehl, Tiago D. Serafim, Serena Doh, Charles S. Grugan, Eva Iniguez, Luana Rogerio, Ronja Frigard, Ranadhir Dey, Pedro Cecilio, Xinglong Gu, Pang-Yen Tseng, Aline Da Silva Moreira, Mahnaz Minai, James Oristian, Hans Ackerman, Steve Brooks, Caroline Percopo, Siu-Ping Ng, Derron A. Alves, Lucas Tirloni, Jennifer M. Anderson, Adriana Marques, Fabiano Oliveira, Shaden Kamhawi, Daniel E. Sonenshine, José M. C. Ribeiro, Mark Hoon, Jesus G. Valenzuela

**Figure S1**

**
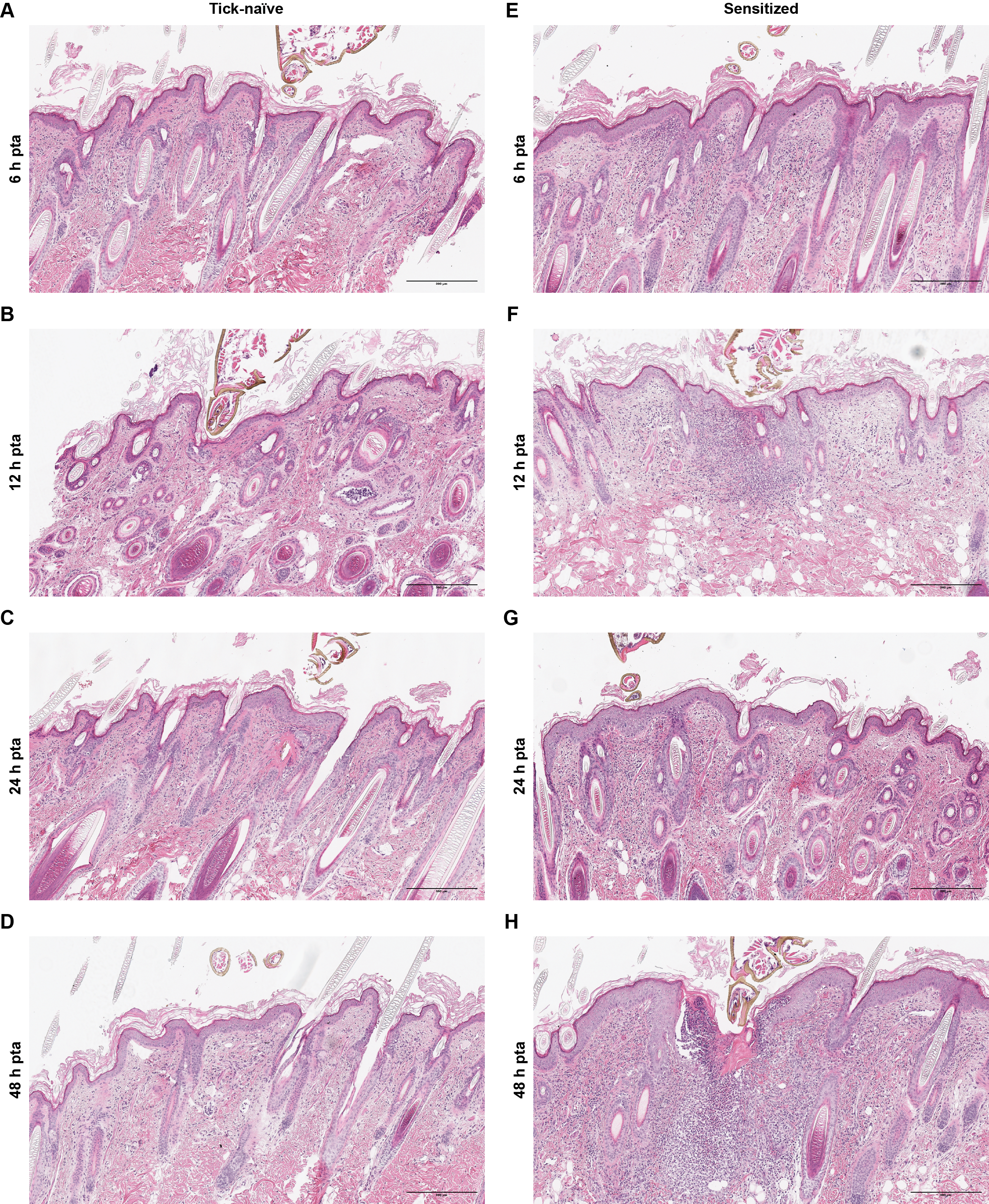
**

**Figure S1.** Rapid cellular infiltration into the tick bite site in sensitized guinea pigs. Representative histological sections (>|5µm|<) stained with H&E showing cell infiltrates at the tick bite sites in tick-naive and tick-sensitized GPs over 48 h pta. Scale bar 300 µ.

**Figure S2**

**
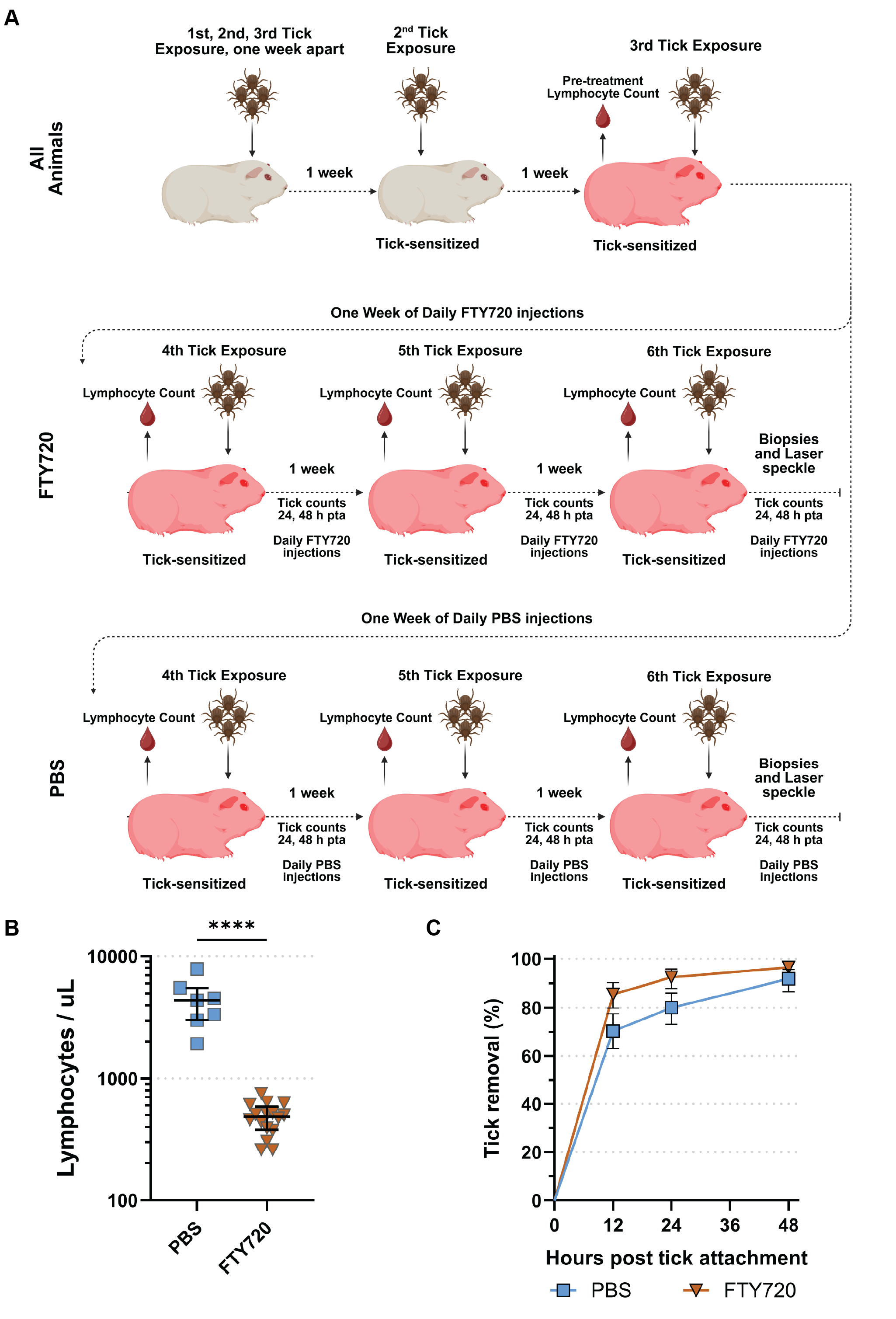
**

**Figure S2**. FTY720 treatment of guinea pigs (GPs) previously exposed to tick bites does not affect tick removal. (A) Experimental design. (B) Scatter Plot: Lymphocyte count per microliter of blood, in PBS-treated (PBS, *N* =7 blood draw samples) and FTY720-treated (FTY720, *N*=16 blood draw samples) GPs. Analyzed by unpaired t-test: ****, *p* < 0.0001. Median shown. (C) Tick removal in PBS-treated or FTY720-treated tick-sensitized GPs after tick attachment (PBS and FTY720: *N* = 4 GPs, *N* = 3 trials). GPs were sensitized 4 times prior to FTY720 treatment. Smoothed, inverted Kaplan-Meyer plot showing probability of tick removal (%) in PBS- or FTY720-treated GPs at 12, 24, and 48 h post tick attachment (pta). Analysis by Peto & Peto modification of the Gehan-Wilcoxon test: *p* < 0.001. Median +_IQR shown. Created in BioRender. Frigard, R. (2025) https://BioRender.com/okq47ut.

**Figure S3**


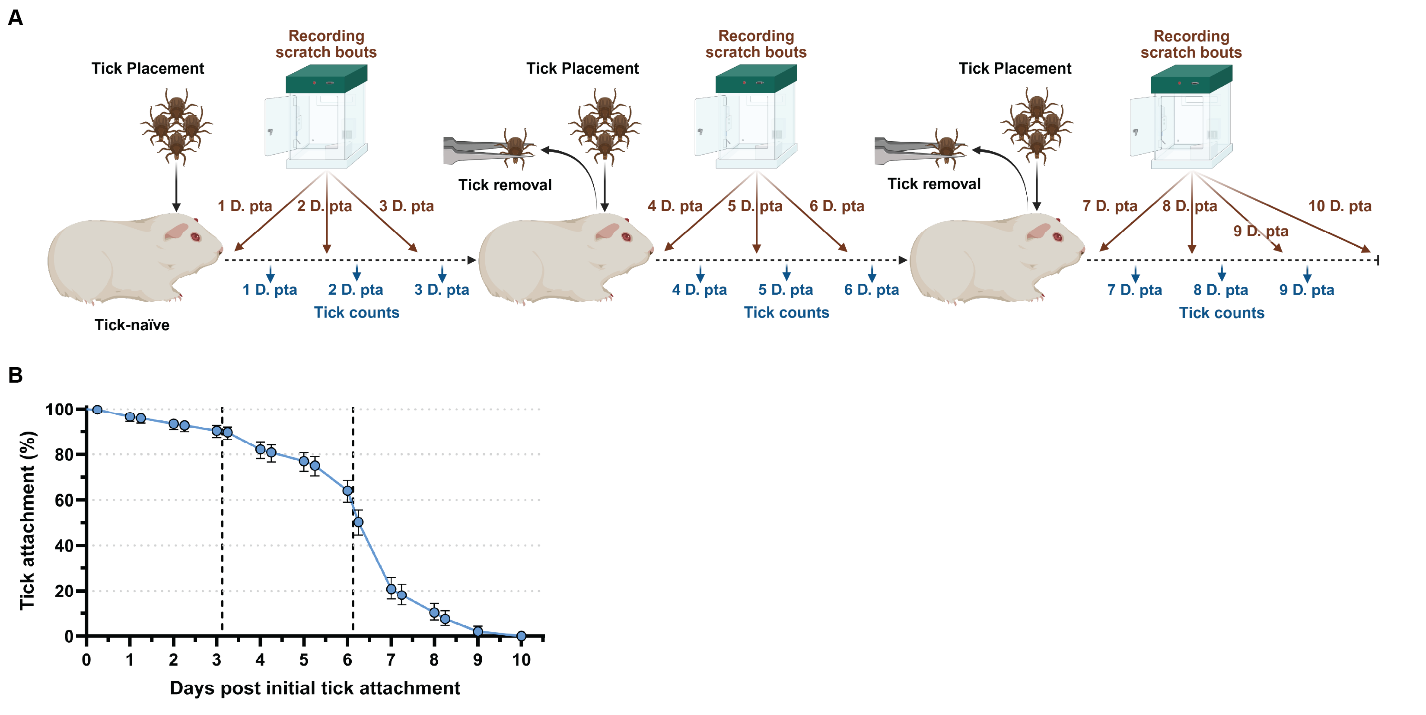


**Figure S3.** (**A**) Experimental design schematic of continuous tick exposure (Created in BioRender: https://BioRender.com/dtivqaj). (**B**) Smoothed Kaplan-Meyer plot showing probability of tick attachment (%) to continuously tick-exposed tick-naive GPs up to 10 days post initial tick attachment (*N*=10 GPs). Dashed vertical lines indicate re-placement of ticks. 95% CI shown.

**Figure S4**


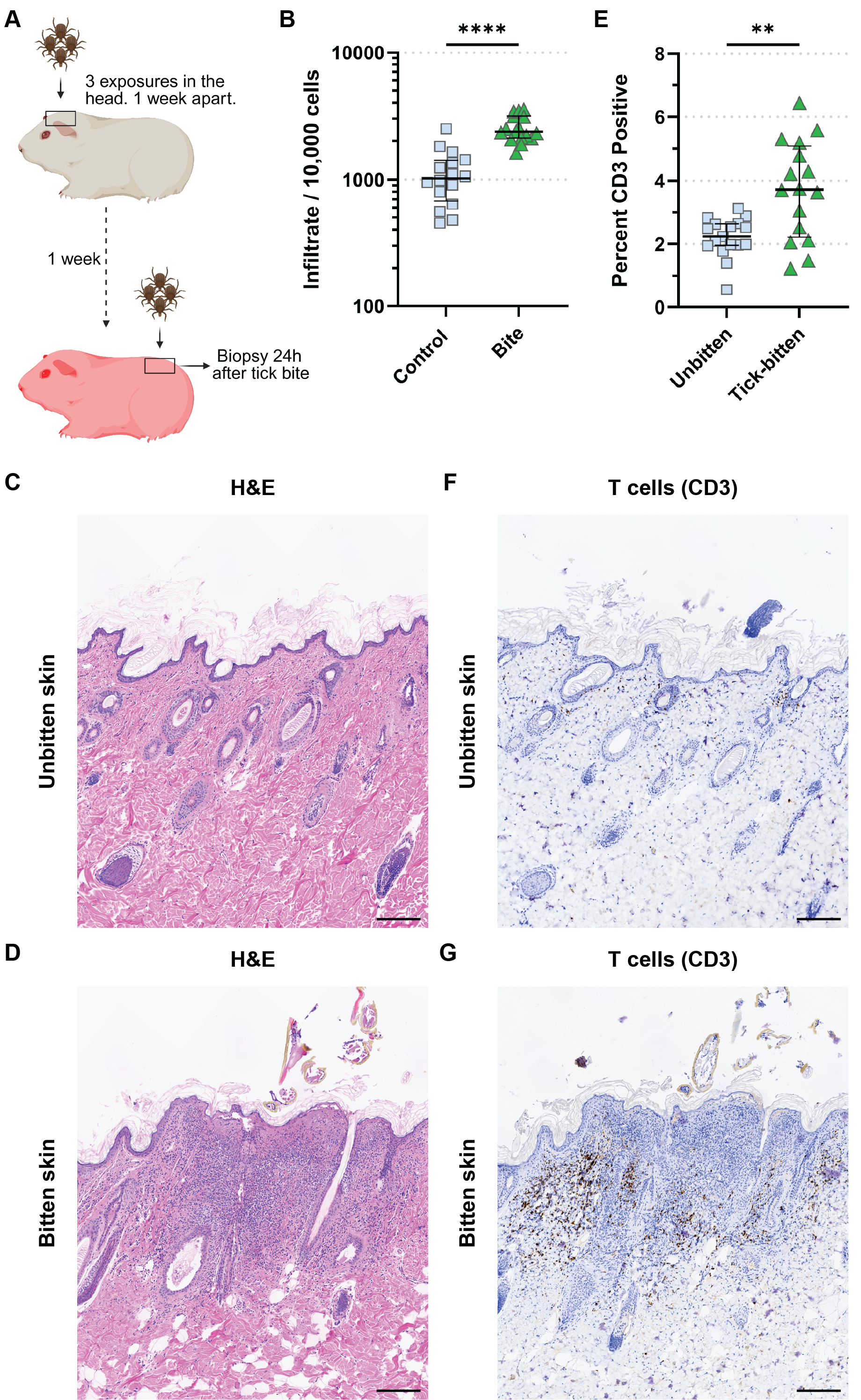


**Figure S4.** Cell recruitment from distant sites of non-bitten and tick-bitten sites after tick re-exposure on tick-sensitized guinea pigs. (**A**) Schematic of GP sensitization with tick placement on head, followed by experimental tick placement on the back and biopsies. Figure S4 Created in BioRender. D. Serafim, T. (2025) https://BioRender.com/637c37o (**B**) Scatter plots, quantification of cellular infiltration in H&E skin cross-sections comparing non-bitten and tick-bitten sites after tick re-exposure on tick-sensitized guinea pigs. (**C, D**) H&E skin cross-sections (>|5µm|<) at 24 h pta at a location (along the spine) distal from the original sensitization location (top of head) (**C**) showing a lack of epidermal hyperplasia and dermal edema with minimal dermal infiltrate at an unbitten site and (**D**) showing significant dermal inflammation and epidermal hyperplasia at the site of tick attachment. (**E**) Scatter plot,

Counts of CD3^+^ (T cells) cells in skin sections of biopsies collected at 24 h pta from a location (along the spine) distal from the original sensitization location. (**F,G**) ICH probed with anti-CD3 (T cells) antibodies of skin biopsies from distal non-bitten (**F**) and tick-bitten (**G)** sites after tick re-exposure on tick-sensitized guinea pigs. Scale bar: 200 µm.
